# Effect of Regular Consumption of a Miraculin-Based Food Supplement on Taste Perception and Nutritional Status in Malnourished Cancer Patients: A Triple-Blind, Randomized, Placebo-Controlled Clinical Trial-CLINMIR Pilot Protocol

**DOI:** 10.3390/nu15214639

**Published:** 2023-11-01

**Authors:** Bricia López-Plaza, Ángel Gil, Adrián Menéndez-Rey, Loan Bensadon-Naeder, Thomas Hummel, Jaime Feliú-Batlle, Samara Palma-Milla

**Affiliations:** 1 Nutrition Research Group, La Paz University Hospital Institute for Health Research (IdiPAZ), 28046 Madrid, Spain; bricia.plaza@idipaz.es; 2 Medicine Department, Faculty of Medicine, Complutense University of Madrid, Plaza de Ramón y Cajal s/n, 28040 Madrid, Spain; 3 Department of Biochemistry and Molecular Biology II, University of Granada, 18071 Granada, Spain; 4 Instituto de Investigación Biosanitaria IBS.GRANADA, Complejo Hospitalario Universitario de Granada, 18014 Granada, Spain; 5 Institute of Nutrition and Food Technology “José Mataix”, Centre of Biomedical Research, University of Granada, Avda. del Conocimiento s/n, Armilla, 18016 Granada, Spain; 6 CIBEROBN (CIBER Physiopathology of Obesity and Nutrition), Instituto de Salud Carlos III, 28029 Madrid, Spain; 7 Medicinal Gardens S.L., 28008 Madrid, Spain; proyectos@baiafood.com (A.M.-R.); loan@baiafood.com (L.B.-N.); 8 Smell & Taste Clinic, Department of Otorhinolaryngology, Technische Universität Dresden, Fetscherstraße 74, 01307 Dresden, Germany; thomas.hummel@tu-dresden.de; 9 Oncology Department, Hospital La Paz Institute for Health Research—IdiPAZ, Hospital Universitario La Paz, 28029 Madrid, Spain; jaime.feliu@salud.madrid.org; 10 CIBERONC (CIBER Cancer), Instituto de Salud Carlos III, 28029 Madrid, Spain; 11 Medicine Department, Faculty of Medicine, Autonomous University of Madrid, Arzobispo Morcillo 4, 28029 Madrid, Spain; samara.palma@salud.madrid.org; 12 Nutrition Department, Hospital University La Paz, 28046 Madrid, Spain

**Keywords:** taste disorders, dysgeusia, neoplasm, chemotherapy, *Synsepalum ducificum*, miracle fruit, malnutrition

## Abstract

Taste disorders are common among cancer patients undergoing chemotherapy, with a prevalence ranging from 20% to 86%, persisting throughout treatment. This condition leads to reduced food consumption, increasing the risk of malnutrition. Malnutrition is associated not only with worse treatment efficacy and poor disease prognosis but also with reduced functional status and quality of life. The fruit of *Synsepalum dulcificum* (Daniell), commonly known as miracle berry or miracle fruit, contains miraculin, a taste-modifying protein with profound effects on taste perception. The CLINMIR Protocol is a triple-blind, randomized, placebo-controlled clinical trial designed to evaluate the regular consumption of a food supplement containing a miraculin-based novel food, dried miracle berry (DMB), on the taste perception (measured through electrogustometry) and nutritional status (evaluated through the GLIM Criteria) of malnourished cancer patients under active antineoplastic treatment. To this end, a pilot study was designed with 30 randomized patients divided into three study arms (150 mg DMB + 150 mg freeze-dried strawberries, 300 mg DMB, or placebo) for three months. Throughout the five main visits, an exhaustive assessment of different parameters susceptible to improvement through regular consumption of the miraculin-based food supplement will be conducted, including electrical and chemical taste perception, smell perception, nutritional and morphofunctional assessment, diet, quality of life, the fatty acid profile of erythrocytes, levels of inflammatory and cancer-associated cytokines, oxidative stress, antioxidant defense system, plasma metabolomics, and saliva and stool microbiota. The primary anticipated result is that malnourished cancer patients with taste distortion who consume the miraculin-based food supplement will report an improvement in food taste perception. This improvement translates into increased food intake, thereby ameliorating their nutritional status and mitigating associated risks. Additionally, the study aims to pinpoint the optimal dosage that provides maximal benefits. The protocol adheres to the SPIRIT 2013 Statement, which provides evidence-based recommendations and is widely endorsed as an international standard for trial protocols. The clinical trial protocol has been registered at the platform for Clinical Trials (NCT05486260).

## 1. Introduction

Dysgeusia, a taste disorder typically characterized by an unpleasant and persistent taste, is often described as metallic, and other taste disorders are common among cancer patients [1]. While dysgeusia can have multiple origins, it is a common side effect for cancer patients who undergo chemotherapy and/or radiotherapy. The recovery from this disorder is a slow and gradual process and can take up to a year after treatment [2]. The incidence of dysgeusia depends on the type of treatment [3], with 45–84% of cancer patients receiving treatment experiencing this side effect [4]. Unpleasant flavors during meals can lead to changes in eating habits, poor nutrition, weight loss, and, ultimately, a decrease in morale, affecting the quality of life [5,6].

To date, the pharmaceutical and supplement industry has been unable to provide effective treatment or strategy for patients suffering from dysgeusia. Zinc supplementation [7,8,9], amifostine [10,11,12], selenium, lactoferrin, and cannabinoids are among the treatments used today, with limited clinically beneficial effects on taste disorders [13,14]. This scenario necessitates new clinical trials to identify effective strategies for controlling dysgeusia and improving the health and quality of life of patients [13,15].

*Synsepalum dulcificum* (Daniell), commonly known as “miracle fruit”, is a plant native to West Africa that contains miraculin, a glycoprotein that can transform sour flavors into sweet ones, making meals more palatable [16,17]. Thereby, miraculin acts as a selective agonist, at an acidic pH, or antagonist, at a neutral pH, of sweet taste receptors, depending on the food pH consumed [18]. Miraculin provides a high sweetness intensity; thus, its consumption could improve the perception of undesirable sour tastes in cancer patients with dysgeusia [19], improving food intake and, consequently, their nutritional status. In December 2021, the European Commission authorized dried miracle berries (DMB) as a novel food in the European Union [20]. DMB, officially cataloged as “dried fruits of *Synsepalum dulcificum*” in the EU, is the freeze-dried extract of the miracle berry’s pulp juice, which is rich in miraculin.

Previous studies have shown the potential of miracle fruit for treating taste disorders. A pilot clinical trial involving 23 patients with dysgeusia derived from chemotherapy treatment demonstrated that the consumption of this berry was safe, and 30% of patients showed an improvement in taste after two weeks of treatment [21]. Another pilot trial conducted two years later on eight patients who experienced taste disturbances after chemotherapy treatment showed that all patients demonstrated improvements in taste, with five patients reporting the disappearance of the metallic taste after supplementation with the fruit [22].

Despite the absence of substantial clinical evidence, prior uncontrolled pilot studies have hinted at the potential of using miracle fruit as a natural dietary supplement, presenting a promising nutritional strategy for managing dysgeusia. The current pilot study aimed to generate clinical evidence on the beneficial effect of the miraculin-based food supplement, containing the miracle fruit extract DMB, on taste disorders. Furthermore, the study sought to explore the interplay between this impact and the enhancement of patients’ nutritional status, employing a rigorous design and methodology. A comprehensive outline of the study’s protocol is provided in this article, adhering to the SPIRIT 2013 Statement (Supplementary Material File S1). This statement, recognized as an international standard for trial protocols (https://www.spirit-statement.org/spirit-statement/, accessed on 20 October 2023), offers evidence-based recommendations for the structure of clinical trial protocols.

## 2. Experimental Design

The CLINMIR study is a pilot randomized, parallel, triple-blind, and placebo-controlled clinical trial. The present protocol clinical trial was registered at http://clinicaltrials.gov, accessed on 20 October 2023, with the number NCT05486260.

### 2.1. Participants and Selection Criteria

A total of thirty malnourished cancer patients and taste disorders will be recruited by the Clinical and Dietary Nutrition Unit and Oncology Service of the Hospital University La Paz (HULP), Madrid (Spain).

The inclusion criteria include patients over 18 years of age with cancer and antineoplastic treatment (chemotherapy and any other treatment such as radiotherapy, immunotherapy, etc., for at least three months) who have a weight loss ≥ 5%, malnutrition assessed by the GLIM criteria [23], and taste disturbances measured by electrogustometry. Additionally, these patients must be starting or be in the first three months of antineoplastic treatment, have a life expectancy greater than 3 months, and be capable of oral intake of food and drinks. Patients also must be be at an appropriate cultural level and understand the clinical study, voluntary participation, and sign the informed consent form.

The exclusion criteria include patients participating in another clinical trial, with enteral or parenteral nutrition, poorly controlled diabetes mellitus (HbA1c > 8%), uncontrolled hypertension or hyper/hypothyroidism, severe digestive toxicity due to treatment with chemo-radiotherapy, severe kidney or liver disease (chronic renal failure, nephrotic syndrome, cirrhosis, etc.), severe dementia, brain metastases, eating disorders, a history of severe neurological or psychiatric pathology that may interfere with treatment, alcoholism or substance abuse, severe gastrointestinal diseases, and an unwillingness to consume the miraculin-based food supplement.

The withdrawal criteria include an inability to tolerate the ingestion of the miraculin-based tablets at any dosage or placebo.

### 2.2. Ethics and Dissemination

The final protocol was approved by the Scientific Research and Ethics Committee of the University Hospital La Paz. The research protocol in version 1 was approved in June 2022 (HULP Code 6164) in compliance with The Ethical Standards of the Declaration of Helsinki about the recommendations guiding physicians in biomedical research involving human subjects. All researchers are to know and follow the ICH Harmonized Tripartite Guidelines for Good Clinical Practice.

Before signing the informed consent form, all subjects will be informed by a study researcher about the study characteristics (verbally and in writing) and what their inclusion in the clinical trial means (File S2). Patients will be informed that they can decide at any time to abandon their participation in the study, notifying their doctor without having to give any reason and without detriment to their usual medical treatment. In this case, they will be asked if their decision is related to any adverse event due to the consumption of the miraculin-based tablets.

All study data will be processed by members of the research team in a database specifically created for this study and dissociated from any data that could identify the patient. The processing of personal data will follow the Spanish Organic Law (Ley Orgánica) 3/2018, of 5 December, and the General Data Protection Regulation of the European Union (EU) 2016/679 of 27 April 2016.

Only the study investigators will have access to the patient’s data with prior authorization from the principal investigator. Once the study is completed, all the data collected in paper format will be archived in the external archive store of HULP and will be kept for the period established by local legislation.

Once the clinical trial has finished, following the provisions of the Spanish Legislation (Real Decreto 1090/2015), the researchers and the promoter will publish the obtained results (positives or negatives). Publication will take place in a publicly accessible scientific journal.

### 2.3. Ancillary and Post-Trial Care

The patients will follow the usual intrahospital clinical practice once the study is completed. The patients will continue to be under the care of their treating physician at the La Paz University Hospital. The present clinical trial is a low-intervention study and no harm from trial participation is expected. 

### 2.4. Dissemination Policy

Trial results will be communicated via publication, reporting in results databases and other data-sharing arrangements. There will not be any publication restrictions. 

### 2.5. Interventions

**Figure 1:**
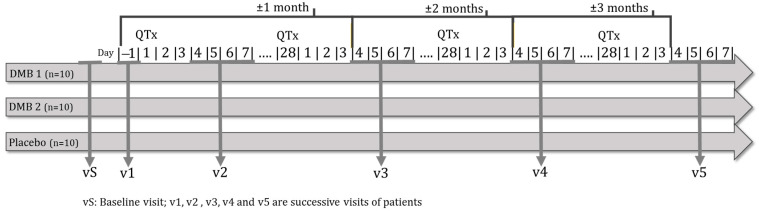
Clinical trial outline.

Malnourished cancer patients in active treatment and with taste disorders will be randomized to one of three arms of the clinical trial. Over 3 months, each patient will dissolve a tablet of the miraculin-based food supplement five minutes before each main meal (breakfast, lunch, and dinner). Each tablet will contain DMB in one of its two doses or a placebo (Figure 1).

Alongside the standard dietary guidelines for cancer patients, participants will be motivated to incorporate acidic foods such as yogurt, vinegar, and lemon drops into their main meals. This approach aims to enhance compliance with the intervention. Each subject will be provided with the precise quantity of the miraculin-based food supplement bottles containing orally dissolving tablets with DMB or placebo necessary until their subsequent visit. Participants will be requested to return all packaging, whether empty or partially used, enabling compliance assessment through a comparison of provided and returned tablet quantities. Compliance will be established if a subject ingests ≥ 90% of the tablets supplied. In instances where the prescribed dose is not adhered to, the principal investigator will terminate the patient’s involvement in the study. Additionally, a dual product consumption monitoring mechanism is incorporated, involving a daily record where patients document the quantity and count of tablets consumed each day throughout the study.

Any pharmacological treatment undertaken during the follow-up period will be registered in an electronic Clinical Research Data Capture (eCRD) platform. Patients will maintain their regular treatment regimen. The use of any medication that does not interfere with the study formulation will be allowed. However, if there is any extraordinary medication, the principal investigator of the study will judge the suitability of the participant’s continued involvement.

### 2.6. Treatments

Each intervention group will be integrated by 10 randomly assigned patients to one of two DMB dosages or placebo. The first arm will have 150 mg of DMB equivalent to 2.8 mg of miraculin + 150 mg of freeze-dried strawberries per orodispersible tablet; the second arm will have 300 mg of DMB equivalent to 5.5 mg of miraculin; and the third arm will contain 300 mg of freeze-dried strawberries per orodispersible tablet as placebo. The three treatments are isocaloric (Table 1). The subjects will be provided with as many tablets as necessary to complete the 3-month intervention period during recurring scheduled visits to the HULP.

### 2.7. Primary Outcome

#### Electrical Taste Perception

Electrical taste perception is evaluated by electrogustometry. Electrical taste testing provides a very accurate means for quantitatively assessing the human taste system [24]. Moreover, functional imaging studies have found that lingual electrical stimulation activates the same brain regions as chemical stimulation [25]. Malnourished cancer patients with taste distortion and consuming the miraculin-based food supplement are expected to improve their taste perception by reducing the taste perception threshold (dB) via electrical stimulation from baseline (v0) to a month after the intervention (v3). The mean of the difference between the scores found between these visits is statistically compared with those found in the rest of the treatments. The evolution of taste perception is also evaluated through the different intervention visits where it is measured (v1, v3, v4, and v5).

The primary sensory system is negatively impacted by a range of diseases and disorders, including early stage cancers [26]. Cancer patients with taste disorders have a 3.36-fold increased risk of malnutrition [27,28]. Malnutrition is related not only to poor prognoses but also to reduced functional status and quality of life [29]. Thus, better food taste perception could enhance food intake and improve malnutrition and the associated risks.

### 2.8. Secondary Outcomes

Clinical trial variables are to be evaluated from the baseline (v0) to the end of the intervention (v5). The mean of the difference between these visits will be compared with those calculated in the other two treatment groups. The evolution of all clinical trial variables will also be evaluated through the different intervention visits where they will be measured (v1, v2, v3, v4, or v5).

#### 2.8.1. Chemical Taste Perception

Chemical taste perception is evaluated by the taste strips test. Taste sensitivity evaluation is necessary for a proper diagnosis and subsequent treatment of taste disorders [30]. Through the taste strips test, it is possible to obtain a total score and identify those patients with taste alterations such as parageusia (incorrect taste identification) and hypogeusia or ageusia (decreased taste sensitivity or lack of taste, respectively) from those with normogeusia (normal perception of taste). Once patients have been diagnosed, the changes that have arisen from the beginning to the end of the intervention and the differences observed with the different treatment groups are to be evaluated.

The taste disturbance prevalence ranges from approximately 20 to 86% [31], which occurs approximately 2–3 weeks after the start of cancer therapy and persists throughout the treatment [32]. These distortions often contribute to difficulties maintaining food intake during treatment [33], thus increasing the risk of malnutrition [28]. As chemical test perception is complementary to electrical taste perception, the consumption of the miraculin-based food supplement might enhance food intake and improve nutrition status due to better food taste perception.

#### 2.8.2. Smell Perception

Taste and smell disorders (TSD) can occur during the course of cancer, and they are associated with anorexia, early satiety, weight loss, malnutrition, and reduced quality of life [34]. Odor food perception is possible through two pathways: the orthonasal pathway, which occurs when odors are perceived directly through the nose, and the retronasal pathway, which occurs when odors are released from food during mastication and reach the olfactory receptor cells in the nose [35]. It has been estimated that 80% of the smell information of a meal is transmitted through retronasal olfaction [36], and it is commonly associated with the sense of taste due to its contribution to the flavor of foods or drinks. However, the orthonasal odor perception pathway is usually more affected than the retronasal pathway in different clinical conditions, with intact retronasal perception in the absence of orthonasal olfactory function [37]. This could be due to the difference in vulnerability between the anterior and posterior olfactory epithelium.

Approximately 60% of patients with disorders in smelling also complain about disorders of taste [38]. These two processes are tightly related; however, they are often described as separate entities due to patients having difficulty identifying them [39]. Since the prevalence of dysosmia is approximately 5–60% in cancer patients undergoing chemotherapy [4], its assessment is essential to complete the taste and smell perception evaluation. In this sense, an improvement in TSD may ameliorate the general food intake and, therefore, the quality of life and nutritional status of patients undergoing active cancer treatment. Orthonasal olfactory function is measured by the Sniffin’ Sticks test, identifying patients with normosmia and hyposmia depending on their age, and is measured in the same visits as the taste tests (v1, v3, v4, v5).

#### 2.8.3. Nutritional Status

Malnutrition is the state resulting from a lack of intake or uptake of nutrition that leads to changes in body composition, reduced fat-free mass and body cell mass, decreased functional capacity, and impaired clinical outcome from disease [40]. This can result from starvation, disease, or aging combined or as separate entities [41]. However, when malnutrition is caused by a concomitant disease, it is referred to as disease-related malnutrition (DRM), where inflammation is usually present [42].

Malnutrition is a condition that is highly prevalent in cancer patients. It is estimated that up to 10–20% of cancer patients die due to malnutrition rather than the tumor [43]. This appears as a consequence of both the tumor and the antineoplastic treatments and negatively affects the quality of life and the effectiveness of treatments. Thus, the assessment of nutritional status in cancer patients is crucial to improve disease prognosis.

Nutritional status is evaluated by the GLIM criteria at the beginning and the end of the intervention (3 months), a period where it is possible to assess the nutritional intervention effect. From the assessment of the nutritional status, a diagnosis of the type of malnutrition presented (moderate or severe) is obtained. Additionally, the nutritional status of the patients will be evaluated at visits 3 and 4 to evaluate the evolution of the patient throughout the clinical trial. For the rest of the variables, the average change occurring from the beginning to the end of the intervention between the different treatments is evaluated.

According to the practical guidelines for clinical nutrition in cancer, nutritional intervention has to lead to increasing oral intake in cancer patients who are malnourished or at risk of malnutrition [43]. In addition to dietary advice, the nutritional intervention is intended to treat symptoms and derangements impairing food intake (e.g., dysgeusia) and offering oral nutritional supplements (ONS) when necessary. In this regard, it is possible that improvement in taste distortions has a positive effect on food intake and, therefore, on the nutritional status of cancer patients.

#### 2.8.4. Morphofunctional Assessment

Morphofunctional assessment consists of a series of evaluations that determine body composition and functionality. These evaluations include classic parameters (anthropometric parameters, biochemical parameters, food intake) and emerging parameters (bioelectrical impedance, nutritional ultrasound, dynamometry, functional tests and biochemical parameters) [44]. The realization of these measurement techniques certainly allows phenotypic categorization and etiologic criteria for malnutrition diagnosis via the GLIM criteria.

Assessment for DRM diagnosis and severity is based on five criteria: three phenotypic criteria (unintentional weight loss, low body mass index, and reduced muscle mass) and two etiologic criteria (reduced food intake or assimilation and inflammation or disease burden) [23]. In addition, each of its evaluations provides information that allows a predictive analysis of nutritional status evolution and prognosis. Therefore, morphofunctional evaluation provides vital information to assess the evolution of nutritional status based on the assigned treatment. Morphofunctional assessment will be measured in the main visits to support monitoring of nutritional status. However, each of these tests individually also provides information on the adequacy of nutritional treatment.

##### Anthropometric Parameters

Even though body weight is not sensitive enough for the early detection of DRM, unintentional weight loss is basic to estimate nutritional requirements, and it is an important screening tool for DRM risk as well as the simplest phenotypic criteria for the diagnosis of malnutrition [45].

Weight losses of 5–10% in the last 6 months or 10–12% in more than 6 months point to moderate malnutrition, while losses >10% in the last 6 months or >20% in more than 6 months point to severe malnutrition [23]. Thus, body weight (kg) and percentage weight loss (%) were measured at the main visits (v1, v3, v4, and v5). Changes that occurred each month are to be evaluated using the mean of the change and compared between treatments to evaluate its evolution throughout the clinical trial.

Because weight loss can be modified by alterations in hydration status (edemas) [46], body mass index (BMI) was calculated. BMI is an anthropometric measure widely accepted for body composition measurement that, even with its limitations (does not measure fat, muscle, or skeletal compartments), is used as a phenotypic criterion for malnutrition diagnoses. BMI values < 20 kg/m^2^ for patients under 70 years and <22 kg/m^2^ for patients over 70 years old point to moderate malnutrition, while severe malnutrition corresponds to BMI values < 18.5 kg/m^2^ in patients under 70 and <20 kg/m^2^ in patients over 70 years old [23]. In cancer patients, weight change and BMI, as well as nutritional intake, are recommended to detect nutritional disturbances at an early stage [43]; therefore, they were evaluated throughout the entire study. BMI was measured at the main visits as well (v1, v3, v4 & v5).

Waist circumference (WC), another anthropometric parameter, together with waist-to-height ratio has been suggested as a stronger predictor for several cancers [47]; they are central adiposity, visceral fat, and chronic disease risk factor indicators and are used as cardiometabolic morbidity and mortality predictors [48]. Recommended waist circumference cutoffs are used to monitor the health risk (>102 cm for men and >88 cm for women) [49] in cancer patients. WC and waist-to-height ratio were measured at the main visits (v1, v3, v4, and v5). Changes occurring in anthropometric parameters are to be evaluated using the mean of the change and compared between treatments to evaluate its evolution throughout the clinical trial.

##### Electrical Bioimpedance

The presence of diseases can produce an increase in body water and/or fat mass without weight loss. Indeed, patients can lose >10% of their body weight over three to six months and have a BMI above or in a normal range [50]. Therefore, body composition measurements are essential for adequate nutritional status assessment. Body composition is to be evaluated via electrical bioimpedance (BIA). This technique estimates the indirect body composition by running a small electrical current through the body and measuring the resistance to body tissues [51]. BIA provides objective information about body compartments (fat-free mass and fat mass) and hydration status (total body water (TBW), extracellular (ECW), and intracellular water (ICW)). Indirect muscle data such as the Appendicular Skeletal Muscle Index (ASMI), Fat-Free Mass Index (FFMI), and Appendicular Lean Mass (ALM) were also measured. From these parameters, it is possible to obtain the body composition of the cancer patient and complete their nutritional assessment. In this sense, low ASMI (<6 in females, <7 kg/m^2^ in males) is used as an indicator of muscle mass loss, one of the phenotypic criteria for malnutrition diagnosis [23].

On the other hand, the raw bioelectric parameters from BIA are to be obtained via direct measurement, without interference of anthropometric factors (weight or age). Through resistance (R) and reactance (Xc), it is possible to obtain the phase angle (PhA). This BIA parameter is an important indicator of cell membrane health and integrity and has been considered a marker of clinical conditions such as cancer [52,53]. A low PhA indicates a poorer nutritional status, as well as impaired cell membranes and muscle function, and may be an important prognostic factor of survival in cancer patients [54]. Indeed, high PhA was significantly associated with lower mortality in cancer patients. A cutoff point was identified using the ROC curves for PhA in cancer patients (≤5.6°) [55]. After adjusting for sex and age, it is possible to obtain the standardized PhA, which is an independent prognostic indicator in cancer patients receiving chemotherapy [56]. Even though the standard cutoff value for standardized PhA has not yet been established [57], changes in the patient over time can be considered changes in nutritional status.

BIA performance is to be carried out at scheduled visits v1, v3, v4, and v5. Changes that occurred in BIA parameters month by month were evaluated using the mean of the change and compared between treatments to evaluate the evolution.

##### Dynamometry

Dynamometry is a functional method to assess muscle strength through handgrip strength [44]. Hand dynamometry is a general marker for nutritional status and is considered a support measure for the assessment of muscle mass, a phenotypic criterion for the diagnosis of malnutrition [23]. Malnutrition implies a reduction in muscle mass, which is reflected in lower performance on functional tests and alterations in body composition [58]. Dynamometry assessment is of special interest because the decrease in muscle strength appears before changes in anthropometric measurements and laboratory parameters are observed. Muscle strength measurement is a useful tool in screening and assessing malnutrition [59] and sarcopenia [60]. Low grip strength is a predictor of longer hospital stays, poor health-related quality of life, and increased functional limitations [61].

Reference values for dominant hand muscle strength to assess DRM have been obtained for the Jamar^®^ dynamometer in a Spanish population [62], and they are to be used in the clinical trial to identify those patients with low grip strength as a support measure in the malnutrition diagnosis and sarcopenia risk. Based on age and sex, patients are to be classified as normal or with low grip strength (<27 kg in males, <16 kg in women) [63]. Hand dynamometry is to be carried out at scheduled visits v1, v3, v4, and v5. Changes occurring in grip strength are to be evaluated using the mean of the change and compared between treatments to evaluate its evolution.

##### Nutritional Ultrasound

Nutritional ultrasound is an emerging technique that uses ultrasound to assess body composition. It is composed of two techniques, i.e., muscle ultrasound (FFM assessment) and adipose ultrasound (FM evaluation). The muscle ultrasound method quantifies muscle modifications in malnutrition and provides information on functional changes [64]. In this sense, the anterior rectum area of the quadriceps can be used as a criterion for malnutrition, and it has been observed that a high rectus femoris cross-sectional area is associated with a decrease in mortality risk in cancer patients. A cutoff rectus femoris cross-sectional area (≤4.47 cm^2^/m^2^) and rectus femoris-*Y*-axis (≤1.3 cm) are identified in cancer patients [55] and used to evaluate changes in muscle composition. The measurement of the rectus femoris muscle of the quadriceps also correlates with force and functional execution or performance tests [65].

Malnourished cancer patients with taste distortion and consuming the miraculin-based food supplement are expected to improve their body composition from baseline (v0) to the end of the intervention (v5). The mean of the difference between the scores found between these visits is to be statistically compared with those found in the rest of the treatments.

##### Functionality Test

Timed-up-and-go (TUG) is a functional test that evaluates physical function through the time a patient takes to rise from a chair, walk three meters away, turn, walk back, and sit down again [66]. This functional test can predict fall risks and mortality [67]. A functional assessment of patients is important for nutritional status evaluation and sarcopenia diagnosis. The final purpose is the functional recovery of the patient, with positive changes in weight, FM, FFM, and functionality [44]. To evaluate the activity and monitor any clinical changes over time, the TUG test is to be assessed at v1, v3, v4 and v5. It used sarcopenia cutoff points for low performance (≥20 s) [60,68]. Malnourished cancer patients with taste distortion and consuming the miraculin-based food supplement are expected to improve functionality from baseline (v0) to the end of the intervention (v5). The mean of the difference between the scores found between these visits will be statistically compared with those found in the rest of the treatments.

#### 2.8.5. Diet

Inadequate dietary intake is an important factor involved in the weight loss and progressive functional decline associated with advanced cancer [69]. Reduced food intake is a well-established indicator of malnutrition that has strong validity. In this sense, less than or equal to 50% of energy requirements for more than one week, any reduction for more than 2 weeks, or any chronic gastrointestinal condition that adversely impacts food assimilation or absorption is considered reduced food intake or reduced assimilation, an etiologic criterion for malnutrition diagnosis [23].

Diet is collected in the 72 h food daily record at v1, v3, v4 and v5, allowing the transformation of food consumption into energy intake, water, macronutrient intake (proteins, fats (total fat, saturated (SFAs), monounsaturated (MUFAs) and polyunsaturated fatty acids (PUFAs), carbohydrates, fiber, and micronutrient intake (vitamins and minerals)). From this information, it is possible to calculate the caloric and lipid profile as well as the coverage of the recommended intakes of the population. Obtained data are to be evaluated using the European Food Safety Authority dietary reference values [70] and the Nutritional Objectives of the Consensus Document of the Spanish Community Nutrition Society [71]. If food intake does not meet energy requirements, a high-calorie, high-protein oral nutrition supplement with fiber, EPA, DHA, beta-glucans, and enriched in L-leucine (no added sugars) will be prescribed.

Dietary habits, diversity and variety will be measured using a quantitative FFQ. To evaluate the overall dietary habits, the consumption frequency of all food items will be categorized as meeting or not meeting the criteria of the dietary guidelines for the Spanish population [72], the food pyramid for the Spanish population [73], and the nutrition goals for the Spanish population [71].

Malnourished cancer patients with taste distortion and consuming the miraculin-based food supplement are expected to improve their dietary intake from baseline (v0) to the end of the intervention (v5). The mean of the difference between intake and food frequency found between these visits will be statistically compared with those found in the rest of the treatments. The evolution of diet will also be evaluated through the different intervention visits where it will be measured (v1, v3, v4, and v5).

#### 2.8.6. Physical Activity

Resting energy expenditure is increased in many cancer patients; however, total energy expenditure appears to be lower because of a reduction in daily physical activity [74]. In cancer patients, total energy expenditure may be estimated from standard formulas for resting energy expenditure and standard values for physical activity level [75]. To calculate the total energy expenditure and energy requirements, physical activity is evaluated. Maintenance or an increase in the physical activity level to maintain physical function, muscle mass, and metabolic pattern is a clinical practical recommendation in cancer patients [43]. Increased physical activity reduces the risk and improves survival of patients in several cancers [76].

To calculate the energy balance, the energy intake obtained in the 72 h daily food record is to be compared with the total energy expenditure calculated from the Institute of Medicine, Food and Nutrition Board [77], plus the energy derived from physical activity obtained through the International Physical Activity Questionnaire [78]. Physical activity will be evaluated at the beginning (v0) and at the end of the intervention (v5). The mean of the difference between intake and food frequency found between these visits will be statistically compared with those found in the rest of the treatments. The evolution of diet will also be evaluated through the different intervention visits where it will be measured (v1, v3, v4, and v5).

#### 2.8.7. Quality of Life

Malnutrition negatively impacts quality of life and oncologic treatment. Thus, nutrition plays a crucial role in heterogeneous cancer care. In patients undergoing (adjuvant) radiotherapy, nutritional support improves nutritional status but also some aspects of quality of life [79]. In patients with advanced cancer, where life expectancy is several months or years, deficits in nutritional status may impair performance status, tolerance treatments, quality of life, and survival [43]. The European Organization for Research and Treatment of Cancer Quality of Life Questionnaire-Core30 (EORTC QLQ-C30) used in the present clinical trial has a strong prognostic value for the overall survival of cancer patients [80]. In cancer patients, taste alterations are present for a long time [81], suggesting that the risk of malnutrition and low quality of life could continue once the treatment is finished.

Quality of life is to be evaluated at the beginning (v1) and at the end of the intervention (v5). The mean difference between these visits will be compared with the rest of the treatments. The evolution of quality of life will also be measured on v1, v3, v4, and v5.

#### 2.8.8. Tolerance and Adverse Events

Nausea, diarrhea, dysphagia, vomiting, constipation, and abdominal pain are gastrointestinal symptoms that are used to measure product consumption tolerance. However, they are also considered supportive indicators that can impair food intake or absorption, an etiological criterion used for malnutrition diagnosis [23]. Symptoms of intensity, frequency, and duration will be collected in a diary sheet throughout the study and evaluated at every visit, and the percentage change between visits will be compared between treatments. Gastrointestinal disorders will be judged using Common Terminology Criteria for Adverse Events (CTCAE) from the National Cancer Institute [82]. These adverse events will be used to discern the severity of food intake or absorption impairment and evaluate tolerance to the nutritional intervention. CTCAE will be evaluated at the beginning (v1) and end of the intervention (v5), and the percentage change between these two visits will be compared between treatments. Evolution will also be measured on v1, v3, v4, and v5.

#### 2.8.9. General Biochemical Parameters

Biochemical parameters such as cholesterol, albumin, or lymphocytes are general health biomarkers that provide indirect information about nutritional status. They have shown a correlation with body protein, nutrient balance, and energy status [44]. Plasma proteins, mainly albumin and prealbumin, are biomarkers of global protein status. Albumin, a visceral protein, shows a high correlation with mortality and morbidity [83]. Prealbumin, which is more sensitive to protein status changes because it has a short half-life (2–3 days) and hydration status does not affect its concentration [84], will also be measured. Albumin and prealbumin concentrations are considered inflammatory biomarkers associated with nutritional risk [85]. Plasma C-reactive protein (CRP) is an acute-phase protein related to inflammation. The CRP/prealbumin ratio has been proposed as a predictor of mortality and hospital stay extension [86]. A CRP/prealbumin ratio > 0.24 is to be used as a cutoff point [87].

In addition, some vitamins, such as vitamins D, B9, and B12, and minerals, such as iron, zinc, and selenium, are evaluated because they are good biomarkers of nutritional status. Complete blood count and leukocyte differential are evaluated, as well as parameters related to glucose metabolism (glucose, HbA1c, insulin), lipid profile (total cholesterol, HDL, LDL, No-HDL, triglycerides), renal metabolism parameters (creatinine, glomerular filtration rate, urate), biochemistry of anemias (ferritin, vitamin B12, serum folate), proteins (albumin, prealbumin, retinol-binding protein, total proteins), parameters related to hepatic metabolism (bilirubin, alanine aminotransferase-ALAT-), and trace minerals and elements (sodium, potassium, chlorine, calcium) on v1, v3, v4, and v5 to monitor the evolution throughout the study. Changes from the beginning (v1) to the end of the intervention (v5) will be compared between treatments.

#### 2.8.10. Specialized Biochemical Parameters

##### Essential Fatty Acids and Polyunsaturated Fatty Acid Status

Malnutrition occurs frequently in cancer patients due to their oncological process and the treatments received, such as surgery, radiotherapy, or chemotherapy. The status of essential fatty acids (EFAs), linoleic acid (LA) (18:2 *n*-6) and α-linolenic acid (LNA) (18:3 *n*-3), as well as their long-chain polyunsaturated derivatives (LC-PUFAs), especially arachidonic acid (AA) (20:4 *n*-6, eicosapentaenoic acid (EPA) (20:5 *n*-3), docosapentaenoic acid (DPA) (22:5 *n*-3), and docosahexaenoic acid (22:6 *n*-3) (DHA), is essential for cellular homeostasis and, in particular, for the maintenance of immune and anti-inflammatory capacity [88,89,90]. It is well known that the EFA and LC-PUFA profile in erythrocyte membranes provides an accurate estimate of body fatty acid status in both healthy subjects [91] and cancer patients [92,93]. In the latter, EFA and LC-PUFA status is altered, usually due to low EFA intake [94]. The improvement of dysgeusia by the miraculin-based food supplement should potentially lead to increased food intake and, therefore, to a substantial improvement in EFA and LC-PUFA status.

##### Biomarkers of Inflammation (Plasma Cytokines)

Cachectic syndrome, characterized by marked weight loss, anorexia, asthenia, and anemia, is inextricably linked to the presence and growth of the tumor and leads to systemic inflammation and undernutrition due to the induction of anorexia or decreased food intake and increased energy expenditure [95]. Systemic inflammation is a physiopathological characteristic of cancer patients. There are multiple origins of inflammation: tumor cells and activated immune cells release cytokines, chemokines, and other inflammatory mediators. In particular, cytokines play a key role as the main humoral factors involved in cancer cachexia, and a large number of them may be responsible for the metabolic changes associated with cancer wasting [96]. Cytokines, namely, interleukin (IL)-1 and tumor necrosis factor (TNF)-α, have been suggested to be involved in cancer-related anorexia, possibly by increasing the levels of corticotropin-releasing hormone (CRH), a central nervous system neurotransmitter that suppresses food intake, and the firing of glucose-sensitive neurons, which would also decrease food intake. IL-1, in particular, has been associated with the induction of anorexia [9] in that it blocks neuropeptide Y (NPY)-a well-known hypothalamic feeding inductor. In addition, IL-6 increases protein degradation in muscle by activating both the nonlysosomal (proteasome) and lysosomal (cathepsin) proteolytic pathways [97]. Tumor-derived factors, other than cytokines, have been proposed as triggers of the wasting process associated with cancer cachexia. Two of these molecules, lipid-mobilizing factor (LMF) and proteolysis-inducing factor (PIF), have been found in tumor-bearing animals and cancer patients [98]. In addition, alterations in the gut barrier in cancer patients and translocation of bacterial lipopolysaccharide (LPS) can contribute to increased inflammation and production of cytokines and chemokines [99].

##### Oxidative Stress and Antioxidant Defense System (ADS)

Oxidative stress occurs because of an imbalance between the production of free radicals and reactive metabolites and the capacity of the antioxidant defense system (ADS) to scavenge them [100]. Reactive oxygen species (ROS) play important roles in the biology of tumorigenesis. Indeed, ROS represent some of the most relevant carcinogenic entities contributing to the different stages of tumor evolution, acting as inducers of both genomic instability and mediators of signaling pathways related to survival, proliferation, resistance to apoptosis, angiogenesis, and metastasis in preneoplastic and neoplastic cells [101]. ROS are produced mainly in five compartments: mitochondria, cytosol, and single-membrane-bound organelles (peroxisomes, endosomes, and phagosomes) [102], but their major activity lies in the mitochondrion, an organelle responsible for cellular aging and ROS production as a natural byproduct of the electron transport chain (ETC) [101,103]. Under the influence of oncogenes, carcinogenic transformation processes are activated, which results in increased ROS and cellular oxidative stress, culminating in the oxidation of proteins, lipids, and DNA [104]. The role of ROS in the pathological process of cancer transcends all its stages and is relevant in the development of metastasis by increasing the invasion and migration capacity of tumor cells. This is a complex process involving the participation of several proteins and transcription factors [105].

When the hydroxyl free radical (●OH) interacts at the level of the puric bases, a modification occurs at the C8 position of the guanine ring, generating a radical that can be oxidized to 8-hydroxy-2′-deoxyguanosine (8-OHdG) or reduced to 2,6-diamino-4-hydroxy-5-formamidopyrimidine (Fapy G) [106]. These resulting products are highly mutagenic and capable of inducing a G:C to T:A transversion, with 8-OHdG being recognized as an important marker of oxidative DNA damage due to its easy detection [107]. ROS have been described to stimulate the production of R-loops of both DNA and RNA that appear during cell replication conflicts and are important inducers of genomic instability; 8-OHdG has been linked to the production of these fragments [108].

Cells can defend themselves against ROS damage due to the existence of several mitochondrial antioxidant defense mechanisms capable of inhibiting free radical synthesis. Among these mechanisms are enzymatic components such as glutathione peroxidase (GPx), superoxide dismutases (SOD), catalases (CAT) and thioredoxins (Trxs) and nonenzymatic components that are produced endogenously such as glutathione (GSH), lipoic acid, coenzyme Q, and some proteins (ferritin, transferrin, and albumin) [109]. Likewise, the role of exogenous antioxidants present in the diet, such as ascorbic acid, tocopherol (vitamin E), carotenes, and phenolic compounds, stands out [100].

Some of these enzymatic components fulfill their antioxidant functions through actions such as catalysis of the dismutation of O_2_^--^ into H_2_O_2_ and O_2_, converting the effects exerted by SOD into essential processes for all cells exposed to O_2_ [110]. GPx catalyzes the reduction of H_2_O_2_ and organic hydroperoxides in the presence of GSH, acting as a hydrogen donor. During this process, GSH is oxidized to glutathione disulfide (GSSG), which is eventually revitalized by a glutathione reductase enzyme [111]. Other catalysts, such as catalases and the Trx system, are responsible for precipitating the decomposition of H_2_O_2_ into water and O_2_ and for reducing the actions of enzymes involved in the cellular redox balance, respectively [112]. On the other hand, nonenzymatic components of the ADS, such as ascorbic acid and tocopherol, remove free radicals synergistically, with ascorbic acid being necessary to generate reduced tocopherol [113]. Like carotenes, tocopherol exerts antioxidant effects by donating electrons to H_2_O_2_, -OH or O_2_^-^ molecules, thereby inhibiting the formation of ROS in the cytoplasm [114]. Frequently, these antioxidant elements become deficient in cancer cells, facilitating the appearance of oxidative stress and the consequent deterioration of the genetic material together with the promotion of other protumoral phenomena such as resistance to apoptosis or alterations in the regulation of the cell cycle [115]. An emerging model suggests that cancer cells increase the production of ROS to activate localized protumorigenic signaling but balance the increased ROS with elevated antioxidant activity to maintain redox balance [101,103].

##### Plasma Metabolomics

Cancer has multiple effects on metabolism that include both rewiring of intracellular metabolism to allow cancer cells to proliferate inappropriately and adapt to the tumor microenvironment and changes in normal tissue metabolism. Metabolomics is a term used for the systematic identification and quantitation of all the metabolic products of a cell, tissue, body fluid, organ, or organism under varying conditions. Indeed, the metabolome of plasma is a dynamic collection of metabolites that represent its net response to current conditions [116]. Metabolomics techniques are used to study metabolic shifts, including changes in metabolite concentrations and disturbed metabolic pathways, in the progression of cancer cachexia and expand the fundamental understanding of muscle loss [117]. In particular, the abundance of paraxanthine is decreased in cancer cachexia patients compared to those without cachexia. These patients undergo radical treatments with radio-/chemotherapy, and high 3-hydroxybutyrate levels are detected at an early stage of the treatment in patients affected by head and neck squamous cell carcinoma cancer. Thus, 3-hydroxybutyrate could be exploited as a fast and sensitive biomarker of malnutrition or cachexia [118]. Overall, 45 metabolites and 18 metabolic pathways have been reported to be associated with cancer cachexia. Using random forest analysis, 15 of these metabolites have been identified as highly discriminating between disease states; within those metabolites, carnosine, leucine, and phenylacetate have been validated [119].

##### Oral and Intestinal Microbiota and Metagenomics

The microbiota or microbiome refers to all microbial organisms that naturally exist within an ecosystem, i.e., part of an organism such as the oral cavity, the intestine, or the vagina. The concept of the microbiome refers not only to the members of the microbial ecosystem but also encompasses their collective functional capacity (metagenome) and activity (metabolome), including interactions within the community and with the host. Indeed, the metagenome is a term used to design the collective genome representative of the many microorganisms existing in a particular ecosystem, and metagenomics is the systematic study of the assemblages of genomes for those microorganisms.

Currently, much attention has been given to the oral microbiome and oral and systemic health [120]. The mouth has a variety of microbes second only to the gut in the complexity of human sites. Differences in the oral microbiome occur with old age, which may be due to oral conditions and diseases, e.g., cancer and other chronic diseases [121]. Many factors contribute to the salivary microbiome profile. In particular, radiotherapy for the treatment of head and neck cancer can cause tissue damage and specifically the salivary glands, causing an alteration in the composition of saliva accompanied by dermatitis, oral mucositis, xerostomia, dysgeusia, dysphagia, and dysbiosis [122]. The latter is related to changes in the quantitative and qualitative composition of the microbiota, mainly increased cariogenic cocci and yeast populations [123]. These changes may lead to altered host–microbe interactions or homeostatic imbalance that can contribute to a disease state, often with inflammation [124]. Recently, the oral microbiome has been suggested to be an explanatory factor for nasopharyngeal carcinoma prognosis. Lower within-community diversity is associated with higher mortality, and certain measures of between-community diversity are related to mortality [125]. Additionally, recent data suggest a potential association of the oral microbiome with mutational changes in oral squamous cell carcinoma [126]. Therefore, modifications in oral biofilms of irradiated patients improving dysgeusia, such as treatment with miraculin, could contribute to better oral health.

Current scientific evidence supports the finding that the composition and modification of the gut microbiome play an important role in the development of many different types of cancer. As an example, alterations in the oral, fecal, and pancreatic microbiome composition have been associated with the etiology and progression of pancreatic ductal adenocarcinoma [127]. Metagenomic studies have reported that the intestinal microbiota can affect not only carcinogenesis but also cancer prevention and that microbes may act through various mechanisms sometimes opposite to each other (e.g., microorganisms are capable of acting as tumor suppressors or, conversely, as oncogenic), giving rise to a complex and bidirectional relationship [128,129]. The gut microbiota has also been found to be associated with extragastrointestinal tumors, such as breast cancer, leukemia, and lung cancer [130]. The intake of the miraculin-based food supplement, particularly in several cancers, e.g., head and neck cancer, would ameliorate dysgeusia and food intake, which in turn could contribute to the improvement of intestinal dysbiosis in cancer patients treated with radiotherapy.

### 2.9. Recruitment and Timeline

The project presentation by the principal investigator to the health personnel of the Oncology Service (HULP), regular visits for nutritionists to the day hospital to explain the project to the patients who attended chemotherapy, recruitment of the Monographic Cancer consultation by physicians of the Clinical Nutrition and Dietetics Unit (HULP), reviewing the patient’s medical history, a recruitment posters in patient waiting rooms are part of strategies for achieving adequate participant enrollment to reach target sample size.

**Figure 2:**
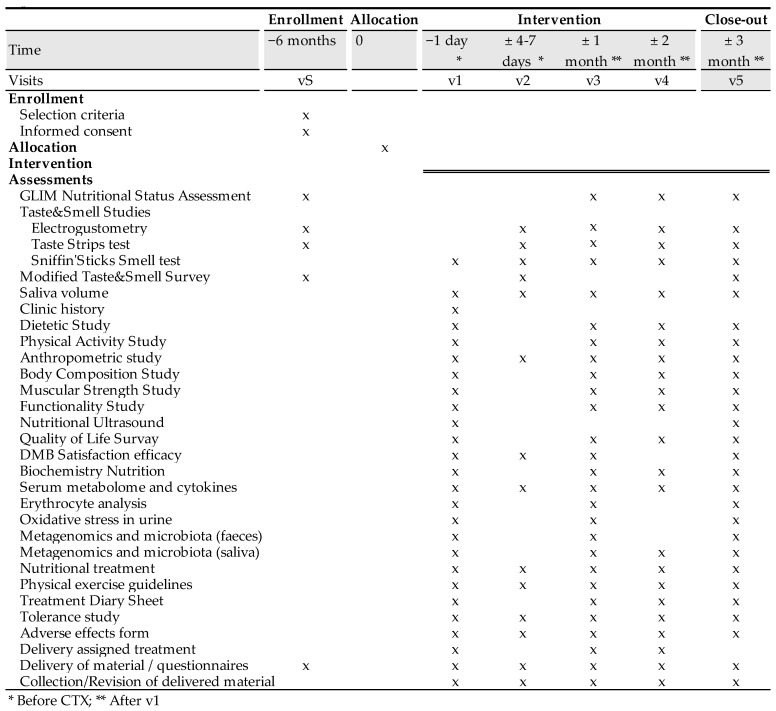
Clinical trial schedule.

The time schedule of enrollment, interventions, assessments, and visits for participants are shown in the schematic diagram (Figure 2).

### 2.10. Sample Size

The sample size is to be established by the researchers owing to the exploratory nature of the study and because there are no previous studies using miraculin-based supplements in cancer patients. At the end of this pilot and to assess the validity of the results, a statistical power calculation will be performed. The obtained results will serve as a basis for establishing the number of participants needed to carry out subsequent efficacy studies.

### 2.11. Assignment of Interventions

Study patients are to be randomized into one of three study arms using computer-generated random numbers listed (in blocks of six) as a method of generating the allocation sequence. This sequence is generated by the Biostatistics Unit (HULP). At the same time, a second list will be filled considering the most prevalent cancer types (head and neck, colorectal, lung, etc.). In this list, the ID patient number is noted in triplicate based on the randomly assigned treatment and cancer type. This second list ensures the balance of each type of cancer in each of the intervention groups. The allocation sequence is provided in a separate document that is unavailable to those who enroll participants or assign interventions. To implement the allocation, the sequence is sequentially numbered in opaque and sealed envelopes by a different researcher who enrolls and assigns participants to interventions. The envelope with the patient’s randomization is to be opened by the nutritionist when the patient signs their informed consent at visit 1.

**Figure 3:**
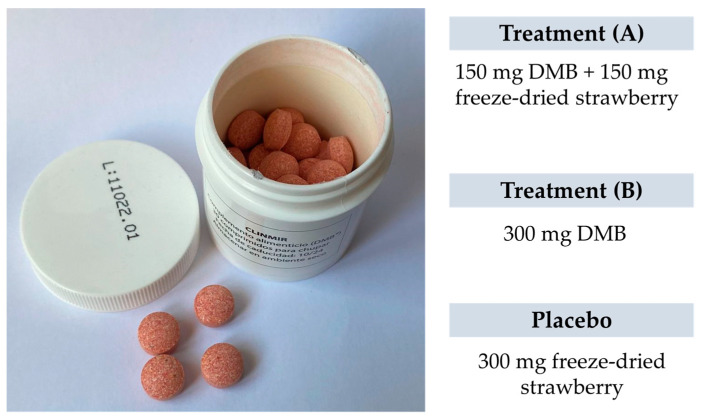
The appearance of the miraculin-based food supplement and placebo.

Researchers, trial patients, care providers, outcome assessors, data analysts, and the promoter are blinded after assignment to interventions. The blinding of the study is achieved by the presentation and identical characteristics of the product to be consumed. The chewable tablets, both the miraculin-based food supplement and placebo, are similar in appearance (Figure 3). These are packaged in white opaque bottles with 30 tablets, uniquely identified by a lot number (L01, L02, L03). The labeling identified the clinical trial name and HULP code, lot and serial number, use and dosage, as well as the expiration date and storage requirements. It is also identified with a unique barcode for tracking.

The chewable tablets are supplied by the promoter Medicinal Gardens S.L. However, they were produced, labeled, identified, and supplied by Activ Vial^®^ from CSP Aptar Technologies, Auburn, AL, USA. This company is the only one aware of the allocation until the opening of the blind once the statistical analysis is completed. To maintain blinding, patients will be identified in the database with the ID number and the assigned lot number. Once the study has been completed, the data file will be verified, any protocol violations will be determined, the statistical analysis will be completed, and the codes will be opened.

### 2.12. Data Collection and Management

The study data will be treated following the referenced confidentiality standards and the quality criteria described above. A specific database will be created in Microsoft Office Standard Excel 2013 (Microsoft® Company) using a standardized database creation process. All data collected in the research data collection will be entered by a member of the research team into the original database. This database will be designed with a double-entry system and filters to prevent and detect any type of inconsistency or error. Data will be incorporated based on the ID patient and visit where the results will be collected (vS, v1, v2, v3, v4, v5). The clinical trial Principal Investigator oversaw supervising the database construction. Databases will be stored in the HULP repository within the closed circuit, and RDC will be stored in the nutritionist’s office in a locked filing cabinet.

During the database creation process, a member of the research team will be in charge of notifying the principal investigator of the presence of missing values. These values will be those that for any reason have not been reflected in the RCD by the patient. The member of the research team indicated the affected cells in the database by coloring them yellow with the word “missing”. Once indicated, the sample size of the affected variable will be adjusted for subsequent statistical analysis. In no case will the patient record be deleted, only the missing value that affects the variable.

After incorporating all the data into the database, they will be duly cleaned, avoiding the appearance of symbols (<, >, etc.) that could interfere with the subsequent statistical analysis. The same member of the research team in charge of creating the original database will be in charge of creating a second database in which alphanumeric coding will be given to each variable to guarantee masking during data analysis. If so needed, new variables such as groupings or sums will be created to facilitate comparison between protocol main visits. When the database is finished, the Principal Investigator will oversee sending the Excel file to the Department of Biochemistry and Molecular Biology II (University of Granada) for analysis.

One of the plans to promote participant retention and complete follow-up is the delivery to the end of the trial of the solidarity cookbook “Cooking with Science against Cancer”, where renowned Spanish chefs designed simple dishes with foods that contain nutrients that are protective against cancer [131].

### 2.13. Statistical Methods

A preliminary study to identify out-of-range values or outliers will be performed. Outliers will be considered those that are greater than the value of the means ± 2 standard deviations. A qualitative descriptive analysis of data will be performed in absolute and relative frequencies and percentages. Quantitative data will be presented as the means ± standard deviations (SD), medians, ranges, percentiles, and percentages. The normality of distribution for each variable will be determined using Kolmogorov–Smirnov and Shapiro–Wilk tests. Levene’s test will be used to evaluate the homogeneity of variances. Parametric or nonparametric tests will be performed depending on whether the variables are normally distributed.

A multivariate analysis of covariance (MANCOVA) for repeated measures will be used to analyze the relationship between a dependent variable and multiple independent variables while controlling for the effects of one or more covariates. A separate analysis of covariance (ANCOVA) on each independent variable controlling for the effects of the covariates will be carried out.

A general linear model (GLM) will be used to model the linear relationship between a dependent variable and one or more independent variables (including continuous, categorical, and binary data). The analysis of the qualitative variables and percentages will be carried out through chi-square (χ^2^) or Fisher’s F analysis depending on the variable sample size.

Specific statistical and bioinformatic software will be used for metabolome, microbiome, and metagenome analyses. This includes PCA, OPLS-DA, analysis of metabolic pathways with mummichog (https://shuzhao-li.github.io/mummichog.org/, accessed on 1 October 2023) and GSEA, PERMANOVA, etc., using the R Project for Statistical Computing. R (https://www.r-project.org/, accessed on 1 October 2023).

Double-sided tests will be applied when needed, and a *p*-value < 0.05 will be considered statistically significant. Data will be analyzed using IBM SPSS Statistics for Windows, version 21.0 (IBM Corp., Armonk, NY, USA) and the R Project for Statistical Computing. R is available as Free Software under the terms of the Free Software Foundation’s GNU General Public License in source code form (https://www.r-project.org/ (accessed on 1 October 2023)).

### 2.14. Monitoring

After carrying out an exhaustive evaluation of the risks and benefits of the study, the establishment of a data monitoring committee was dismissed. This is a pilot clinical trial for the establishment of future study parameters; it has a small sample size, is not a long-term study, and has a low-complexity design [132]. In this sense, it has been established that it is a clinical trial with a low level of intervention, and it has a favorable safety profile and a very low risk of serious adverse effects. Additionally, although the parameters set to be measured are extensive, the collection and data analysis are relatively simple. However, periodic information on the clinical trial status will be communicated to the HULP Ethics Committee.

However, the stakeholders involved will meet every week through an online call to update on the clinical trial progress and any eventuality that may arise during the study. This commission is made up of the principal investigator of the study who summarizes the status of the study, raises the drawbacks, possible solutions to the problems, and supervises the proper development of the study; the study coordinator who presents the patients recruited, in follow-up, discharges, etc., and manages the consumables used; the project coordinator, who stipulates the next steps of the project (publications, communications to congresses, management of body samples); and the promoter, in charge of supplying the study products and providing the consumables material of the study.

The clinical trial medical team will oversee evaluating, reporting, and managing possible adverse events and other unintended effects. In this case, it will be the principal investigator, a physician by profession, who will make the final decision to end the clinical trial.

## 3. Materials and Equipment

### 3.1. Olfactory-Gustatory Tests

#### 3.1.1. Electrogustometry

The threshold for an electric-induced taste stimulus (taste acuity) was measured using an electrogustometer (SI-03 Model, Sensonics International, Haddon Heights, NJ, USA). The electric stimulus is applied with a monopolar electrode (a single, flat, circular stainless-steel stimulus probe) placed on the tongue between the tip and behind the *sulcus medianus linguae*. The electrogustometer produces low-amplitude stimuli of a predetermined duration (0.5 s). All patients were instructed not to eat or drink for an hour before beginning the test. First, a 30 dB (256 μA) stimulus is administered to test whether the subject is capable of recognizing electrogustometric stimuli. Once the threshold is checked, the stimulation starts at the zero-stimulus amplitude, and increasingly stronger stimuli are presented until the patient identifies the stimulus. To measure detection thresholds, the two-down one-up forced-choice single staircase procedure and a stimulus-response staircase are used. If the threshold for stimulus perception is not detected, the next higher- and lower-strength stimuli are presented to the patient. The electric threshold scores are measured at the area of the fungiform papillae on both sides of the tongue. Thresholds are measured in a randomized sequence chosen by the researcher.

The test starts with the 0 dB (8 μA) configuration. If the patient fails to identify the stimulus (−) or (+ −), the intensity is increased (6 dB); if the patient succeeds in both measurements (+ +), the intensity is lowered (−6 dB). The same procedure is repeated at the new intensity, and the same criterion is maintained. If the patient fails (−) or (+ −), the intensity is increased, and if they succeed (+ +), the intensity is lowered. When the first negative point is reached, it is taken as a reference to start the analyses. From this point on, a total of 4 points need to be acquired: one positive (+ +), one negative (−) or (+ −), another positive (+ +), and another negative (−) or (+ −). This is represented in the template as a staircase. Once these four points have been obtained, an average of the decibel (dB) values obtained should be taken.

According to the guidelines of the German Society for Olfactology and Gustology, threshold differences between the left and right sides greater than 7 dB are considered abnormal [133].

#### 3.1.2. Taste Strips Test

Taste strip tests are used to measure chemical taste perception. Based on taste-impregnated filter paper strips, the taste strip test is a validated examination method to determine the ability to taste [30,134] (“Taste Strips”, Burghart Messtechnik GmbH, Holm, Germany). Four different taste strips (sweet, sour, salty, and bitter) in four different concentrations (sweet: 0.4, 0.2, 0.1, 0.05 g/mL sucrose; sour: 0.3, 0.165, 0.09, 0.05 g/mL citric acid; salty: 0.25, 0.1, 0.04, 0.016 g/mL sodium chloride; bitter: 0.006, 0.0024, 0.0009, 0.0004 g/mL quinine hydrochloride) are used. These taste strips are presented successively to the cancer patients in a pseudorandomized manner (Test sequence I: U-D-P-L-H-U-G-C-O-K-V-F-B-J-N-A-E-I-M; Test sequence II: U-H-L-P-D-U-K-O-C-G-V-N-B-F-J-E-M-I-A). Umami is not tested as a descriptor because it is not widely used in Spain. In addition to the impregnated strips, strips with no taste (blank U+V) are offered at the beginning of the test.

One hour before testing, subjects are asked not to eat or drink anything except water, not to smoke, and not to brush their teeth. For the assessment of whole-mouth gustatory function, the strips are placed on the tongue, and the mouth is closed for 10 s (the tongue may be moved). Once the strip is removed, the participant is asked to identify the taste. When a taste is not identified, participants are forced to guess. After each strip, the patient’s mount is rinsed with water to cleanse the palate.

Each correct answer yields one point with a maximum score of 16 points (4 concentrations of each of the 4 basic taste qualities). Blanks are not counted during the evaluation. At a score below the threshold value of nine, hypogeusia can be assumed regardless of age. Complete ageusia can be assumed if the highest concentrations of all four qualities are not detected. The total taste performance and the performance of each taste quality can be evaluated separately, for example, hypogeusia (total score = 7) with partial ageusia for sweet (“no perception of sweet”).

#### 3.1.3. Sniffin’ Sticks Smell Test

**Figure 4:**
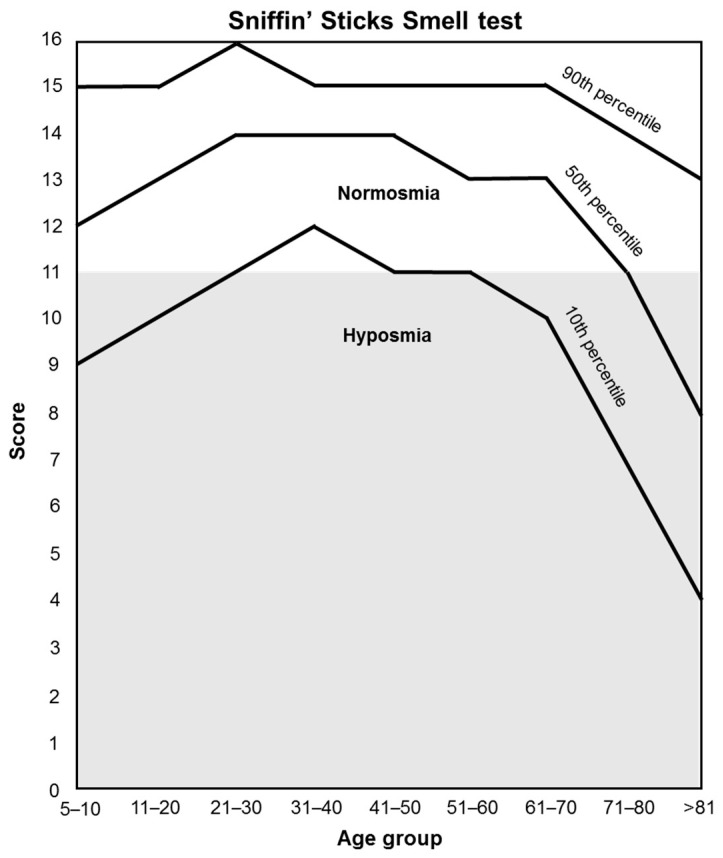
Orthonasal olfactory function classification.

Orthonasal olfactory function (smell perception) is measured by the “Sniffin’ Sticks” test [135], which is based on odor-containing felt-tip pens (“Sniffin’ sticks” Burghart Messtechnik GmbH, Germany). This test consists of a total of 16 odors that are presented to the patient to be identified. The patient will not consume food, drinks, or cigarettes 15 min before testing, and tap water is permitted. For each odor pen, a choice card with 4 choices is provided (e.g., orange, strawberry, blackberry, pineapple). Patients have to choose the term that best matches their olfactory perception. Each is uncovered and held 2 cm from the tip of the nose for 3–4 timed seconds. When an odor is not identified, participants are forced to guess. The 16 pens are presented to the patient one after another at an interval of approximately 30 s. The score sums up all correct answers. Test results are used to differentiate between normosmia and hyposmia depending on the age of the patient (Figure 4).

### 3.2. Malnutrition Criteria

The nutritional diagnosis of malnutrition is established through the GLIM criteria (Global Leadership Initiative in Malnutrition), which classify nutritional status based on phenotypic and etiological criteria [23] (Table 2).

Reduced muscle mass is a phenotypic criterion with strong evidence to support its inclusion in the GLIM consensus criteria. GLIM refers to recommendations from the European Working Group on Sarcopenia in Older People (EWGSOP) [60]. Bioelectrical impedance analysis (BIA) and the body composition method are used to evaluate reduced muscle mass, and hand grip strength functional assessment is considered a supportive measure.

To assess the reduced food assimilation, gastrointestinal symptoms as supportive indicators that can impair food intake or absorption (vomiting, dysphagia, constipation, nausea, diarrhea, or abdominal pain) are considered. They are used as a form of clinical judgment to discern severity based on the degree to which intake or absorption is impaired. Symptom intensity, duration, and frequency are noted.

Inflammation status, major infection, trauma, and acute conditions associated with mild to moderate inflammation are considered. Transient inflammation of a mild degree is not considered a threshold for this etiologic criterion. Chronic disease-related is associated with malignant disease, chronic obstructive pulmonary disease, congestive heart failure, chronic renal disease, or any disease with chronic or recurrent inflammation. Indeed, C-reactive protein (CRP) is used as a supportive laboratory measure of inflammation.

The GLIM criteria require at least one phenotypic criterion and one etiologic criterion to diagnose malnutrition. Malnutrition seriousness is determined based on the phenotypic criteria classifying it as moderate or severe malnutrition [23] (Table 2).

### 3.3. Morphofunctional Studies

#### 3.3.1. Anthropometric Parameters

Anthropometric variables are taken using standard techniques, following the international norms established by the WHO [136]. Body weight is measured using a clinical digital scale (capacity 0–150 kg), preferably in the morning after 12 h of fasting and after evacuation to obtain the most stable values. When it is not possible, the time of day is noted. Height is measured with a height meter with an accuracy of 1 mm (range, 80–200 cm). This measure requires the subject to stand with their feet together and their heels, buttocks, and upper back in contact with the scale. The head should be positioned in the Frankfurt plane (the lower edge of the eye socket is in the same horizontal plane as the upper protrusion of the ear’s tragus) to obtain the highest point of the skull [137]. The patient is instructed to take and hold a deep breath, and while keeping the head in the Frankfurt plane, the measurement is taken at the end of a deep inhalation. Waist circumference is taken at the narrowest level, between the lower costal edge (10th rib) and the iliac crest. If there is no obvious minimum waist, the measurement is taken at the midpoint. The tape is readjusted to ensure that it does not slip or conform excessively to the skin. The subject should be breathing normally, and the measurement is taken at the end of an expiration (at the end of tidal volume) [138]. Body mass index (BMI) is determined using the following formula: [weight (kg)/height (m)^2^]. The relationship between the waist circumference (cm) and the height (m) of the individual is determined to obtain the waist–height index.

#### 3.3.2. Bioelectrical Impedance Analysis

Body composition is evaluated using a multifrequency BIA (Bioelectrical Impedance Analyzer), the EFG ElectroFluidGraph^®^ (Akern S.R.L., Pontassieve, Florence, Italy). Standardization of the BIA technique is a fundamental aspect of the validity of the estimates. In this study, the standard four-pole technique is used [139]. The conditions to reduce variations and guarantee the reliability of the measurement before performing the BIA are as follows: patient in a relaxed state, supine position on a nonconductive surface with bare feet and minimal clothing, limbs abducted at 45°, no metal accessories (earrings, chains, bracelets, etc.), adequate room temperature, fasting for more than 2 h and without consuming alcohol, coffee, caffeinated soft drinks or chocolate in the 24 h before the study; no strenuous exercise in the previous 24 h, preferably not being in the menstrual period, and not coinciding with the 3 days that precede the same.

Subsequently, BIA electrodes are placed on the skin’s surface, on the right hand and on the right foot, according to a standard protocol. The inductors are positioned at a distance in the metacarpal (on the back of the hand between the knuckles of the index and middle fingers) and metatarsal (top of the foot at the base of the toes) line through surface electrodes. The sensors (black clamps) are attached via electrodes to the wrist and ankle joints and therefore inside the electric field. Once placed, a low constant electrical current is passed through the electrodes. The current is at a frequency of 50 kHz and a voltage of 800 millivolts. BIA equipment measures the resistance encountered by the electrical current as it passes through the body. This resistance is then used to estimate the body composition (fat mass (kg), free fat mass (kg), body cell mass (kg), total, extracellular, and intracellular water (L), hydration (%), ASMM (kg), SMI (kg/m^2^)) and other parameters (resistance (R), reactance (Xc), impedance (Z), phase angle (PhA), and standardized PhA), taking into account the height, weight, age, and sex of the patient through a mathematical algorithm. To calculate the ASMM index, the following formula was used: ASMM (kg)/height (m)^2^.

A vectorial analysis (BIVA) of the data was carried out to determine the nutritional and hydrated status.

#### 3.3.3. Dinamometry

A dynamometer was used to measure patient strength and to determine muscle functionality in both hands. Strength was assessed by using a hand hydraulic dynamometer (Jamar^®^, Performance Health Supply Inc., Nottinghamshire, UK) with an adjustable handle, five grip positions (35–87 mm), a hydraulically sealed dual scale (pounds and kilograms), and an isometric grip force indicator (0–90 kg). The maximum force indicator remains after each reading until it is reset. The Jamar dynamometer has been shown to have high reliability and validity for measuring grip strength [140].

To ensure accurate and consistent results, the following standardized procedures are followed [141]. The patient has to rest for at least 5 min before. Any jewelry or objects that may interfere with the grip are removed. The dynamometer handle is adjusted to fit the subject’s hand size. The red peak-hold needle counterclockwise has to be zero. Patients are instructed to sit upright in an individual sitting position with their shoulder adducted and neutrally rotated, elbow flexed at 90°, wrist between 0 and 30° dorsiflexion and between 0 and 15° ulnar deviation, and forearm resting on a flat surface. The dynamometer handles parallel to the fingers. The patient is instructed to squeeze the dynamometer handle as hard as possible using their dominant hand for 5 s. The maximum force in kilograms displayed on the dynamometer is recorded. The grip strength measurement was repeated three times with a 30 s rest period between each measurement to obtain an average score. Scores within two standard deviations of the mean are considered within normal limits. For their interpretation, patient hand grip strength is compared to reference values based on age, sex, and body size for the Spanish population [62]. Grip strength is a reliable indicator of overall muscle strength and is correlated with daily living functional abilities.

#### 3.3.4. Nutritional Ultrasound

The Nutritional Ultrasound^®^ methodology is based on the technical considerations and standardization carried out in Spain [64].

Muscle ultrasound technique. Ultrasound analysis measures muscle architecture parameters [142]. The present study focuses attention on the *rectus femoris* muscle of the quadriceps due to its correlation with strength and functional execution or performance tests [65].

Grayscale ultrasonography with a linear transducer with a coefficient of variation of 1.3% is used (Mindray Z50 Serie, Shenzhen, China). The measurement of the loss of muscle thickness is carried out using a linear transducer with high-frequency sound waves (1–10 MHz). This measurement includes the *vastus medialis* and *rectus femoris* muscles.

Twenty-four hours before the start of the procedure, the patient is to avoid exercising or performing strenuous activities. The patient being tested is to wear a gown to allow easy access to the muscle. First, the patient is placed in the supine position with the legs relaxed and extended at a 45° angle. Ultrasound conductive gel is applied to the skin over the muscle. The ultrasound probe is placed on the skin and moved over the muscle to obtain the muscle tissue images. Ultrasound sends high-frequency sound waves into the muscle tissue, which bounce back to the probe and create an image on the screen. A transverse image of the right leg was obtained. With the transducer at 90 degrees to the thigh, two measurements are taken at different thigh heights. The image is taken in the lower third of the imaginary line connecting the anterior superior iliac crest and the upper border of the patella once the anterior rectus muscle is located. Data obtained by muscle ultrasound are axes (X (cm) and Y (cm)), circumference (cm), and area (cm) of the anterior rectus muscle and subcutaneous adipose tissue (cm).

The adipose ultrasound technique is useful for evaluating the fat mass compartment: subcutaneous (superficial and deep-layer) and preperitoneal visceral adipose tissue of the abdomen. With the patient in the supine position, the transducer is kept perpendicular to the skin placed between the xiphoid process and the umbilicus along the linea alba. The skin is touched as lightly as possible with the transducer so that the fat layers are not compressed. Images are taken during expiration in a transverse plane with a variable probe depth of 4–10 cm, perpendicular to the skin. Superficial and deep layers are differentiated. Visceral adipose tissue is measured using the Hamagawa technique [143]. In the transverse position, the distance between the limit of the parietal peritoneum and the linea alba on the inside at the junction of the two *rectus abdominis* muscles is measured. Data obtained by abdominal ultrasound are total adipose tissue (cm), superficial adipose tissue (cm), and preperitoneal visceral adipose tissue (cm).

The superficial subcutaneous fat layer is related to energy reserve, and a deep subcutaneous fat layer may play a role in neuroendocrine regulation via the secretion of adipokines; preperitoneal visceral adipose tissue is a visceral ectopic tissue (hepatic steatosis equivalent), and other pathological adipose tissues and visceral deposits are related to metabolic manifestations such as diabetes or atherosclerosis [44].

#### 3.3.5. Timed up and Go Test

The up-and-go test is a simple clinical assessment tool used to evaluate a patient’s ability to perform functional tasks (walking and turning), balance ability, and coordination. During the test, patients are asked to rise from a seated position (back supported and their arms resting on the armrests), walk a distance of 3 m, turn around, walk back to the chair, and sit down again. The time taken to complete the task is recorded. When the time required to complete the up-and-go test is ≥20 s, the patient is considered frail [60,68].

### 3.4. Surveys

#### 3.4.1. Taste and Smell Survey

A modified version of the Taste and Smell Survey (TSS) [144] is used to explore the prevalence, characteristics and severity of taste and smell changes since becoming ill. This TSS has been used before in the oncology setting [145,146], including advanced cancer [144]. The TSS responses generate a chemosensory complaint score. It consists of 12 items, 7 of which are related to taste and 5 related to smell. One point is awarded for each reported complaint. An additional point is awarded when it is mild/moderate or rarely/sometimes and a further 2 points if it is severe/disabling or often/always. Taste changes are scored from 0 (no change) to 10 (multiple severe changes) and smell changes from 0 (no change) to 5 (multiple severe changes). A combined chemosensory complaint score of 0 to 15 points is calculated. Two open questions (Questions 10 and 12) allow patients to describe how taste and smell disturbances impact their quality of life. Question 6 is also unscored.

#### 3.4.2. Food Daily Record

For the quantitative assessment of the intake of energy and nutrients, a daily food record is used, following the recommended guidelines [147]. Foods, drinks, dietary supplements and preparations consumed for three days (one of which must be a holiday) before the consultation are recorded. The information obtained is structured in mealtimes (breakfast, mid-morning, lunch, mid-afternoon, dinner, and other meal moments). Patients are asked about the meal place (home, work, restaurant, etc.), food consumption time, and time spent (start and end time meal is registered). Patients provide a detailed description of food consumption (ingredients, method of preparation, brands, type). Subjects are instructed to record the weight of the food consumed or, if this is not possible, to record household measurements (spoonfuls, cups, etc.). At each visit, all records are thoroughly reviewed by a nutritionist in the presence of the patient to ensure that the information collected was complete. To facilitate the process, the nutritionist uses the “Photo guide of common portion sizes of Spanish foods”, which includes 12 food groups, 204 foods commonly consumed by the Spanish population, and 944 photographs [148].

The energy and nutritional content of the foods and beverages consumed are calculated using DIAL software (Alce Ingeniería, Madrid, Spain https://www.alceingenieria.net/nutricion.htm, accessed on 1 October 2023).

#### 3.4.3. Food Frequency Questionnaire

Dietary patterns are assessed using a semiquantitative 137-item food frequency questionnaire (FFQ) previously validated in Spain [149]. The questionnaire is based on the most common portion sizes (weight (g) and volume (mL)) and food groups in the Spanish population that are multiplied by the consumption frequency for each food. Frequency categories included never or almost never; 1–3 times a month; 1, 2−4 or 5−6 times a week and 1, 2−3, 4−6, >6 times a day. Although the FFQ indicates a range of consumption for each food, in the present study, the exact frequency of consumption is specified (2 times a week, 3 times a week, etc.). The information collected from the FFQ is obtained face-to-face with the patient by a trained nutritionist. The FFQ assesses the individual’s diet through nine food categories and 150 items: milk and milk products; eggs, meat and fish; vegetables and vegetables; fruit; legumes, cereals and potatoes; oils and fats; pastries and pastries; sauces, fried foods, snacks, sugars and salt; and drinks. It also includes a special category about dietary supplement/product consumption.

#### 3.4.4. Physical Activity

To assess physical activity patterns, the short version of the International Physical Activity Questionnaire (IPAQ) validated in the Spanish population is used [78]. This questionnaire consists of seven questions that explore physical activity patterns over the past 7 days. The questionnaire is divided into two sections. In the first section, patients are asked to describe the type, frequency, and intensity of activities performed in four areas (at work, at home, during transportation, and during leisure time). Questions are presented on a frequency scale (days per week) and duration (minutes per day). In the second section, patients are asked to indicate the time spent sitting during a typical day of the week. Questions are presented on a time scale (hours per day). Once the questionnaire is completed, a total physical activity score is calculated based on the duration and frequency of reported activities. The results provide quantitative [total energy metabolism rate (MET) per minute/week] and qualitative (low, moderate, or vigorous activity) information on physical activity.

To calculate the quantitative physical activity, time dedicated to each activity is taken into account (8 MET for vigorous activities, 4 MET for moderate activities, and 3.3 MET for walking) by applying the following equation: Total MET (minutes/week) = MET × minutes/day × days/week.

To obtain qualitative information on physical activity, the following assumptions are considered. Low Activity (no activity is recorded or some activity is reported but not enough to fall into moderate activity), Moderate Activity (≥3 days of vigorous activity at least 20 min/day or ≥5 days of moderate activity and/or walking at least 30 min/day or ≥5 days of any combination of walking, moderate or vigorous activity or ≥ 600 total MET min/week), Vigorous Activity (≥3 days/week of high activity and at least ≥1500 MET min/week or ≥7 days/week of any combination of walking, moderate or vigorous activity + ≥3000 MET min/week). Time sitting (h) or walking (min) is also recorded.

#### 3.4.5. Quality of Life

Quality of life (QoL) is evaluated using the EORTC QLQ-C30 questionnaire for cancer patients validated in Spanish [150]. The questionnaire consists of 30 questions referring to the previous week. This is formed by 5 functional scales (daily activities and physical, emotional, cognitive, and social functioning), 3 symptomatic scales (fatigue, pain, nausea, and vomiting), 1 overall health scale, and 6 questions about dyspnea, insomnia, anorexia, constipation, diarrhea and economic impact. Values between 1 and 4 points are assigned (absolute (1 point), little (2 points), quite (3 points), and a lot (4 points)). The last two items (29 and 30) are given a score from 1 to 7, with 1 being terrible and 7 being excellent.

Scores obtained are standardized from 0 to 100 points to determine the disease impact for each scale. To score the scales, first, the average of the items that contribute to the scales (raw score, *RS*) is estimated. *RS* = (*Q*_1_ + *Q*_2_ +…+ *Q*_n_)/*n*. Then, a linear transformation is used to standardize the RS using the following formulas: Functional scales = [1 − (RS − 1)/range)] × 100, Symptom scales/items and global health status (QoL) = [(R − 1)/range] × 100. The range is the difference between the maximum possible value of *RS* and the minimum possible value. A high score on the global health status and functional scales indicates a better quality of life, while a low score on the symptoms scale indicates a decrease in quality of life.

#### 3.4.6. Miraculin-Based Food Supplement Perception Efficacy

A visual–analog scale (VAS) was designed by the researchers to obtain information about the miraculin-based food supplement consumption patient perception efficacy. The questionnaire includes 5 questions to answer on a 10 cm scale, where 0 means not at all or very bad and 10 means very good or very effective. The questions included are as follows: Do you notice a food taste change after consuming the product? Does food taste better to you? Does it allow you to eat more food? What is your opinion about the product? Are you satisfied with the effectiveness of the product? Does the administration of the product seem adequate to you?

#### 3.4.7. Tolerance and Adverse Events

A diary sheet is given where the patient writes down the possible adverse events, symptoms presented, symptoms date (beginning and end), intensity (mild, moderate, intense), a possible relationship with the product (yes, no, it is possible) and behavior adopted. On every visit, the patient is also asked about any other discomforts not written down because they are considered light and related to product consumption. Gastrointestinal disorders such as abdominal distension, abdominal pain, nausea, regurgitation or gastroesophageal reflux, vomiting, constipation, diarrhea, and flatulence are defined and recorded based on Common Terminology Criteria for Adverse Events (CTCAE) from the National Cancer Institute [82]. These adverse events are classified as Grade 0 (not described), Grade 1 (mild), Grade 2 (moderate), Grade 3 (severe), Grade 4 (mortality risk), and Grade 5 (death associated with an event).

#### 3.4.8. Blood Pressure and Heart Rate

Trained personnel measure blood pressure (BP) and heart rate in the right arm in triplicate with a validated semiautomatic oscillometer (Omron HEM-705CP; Hoofddorp, The Netherlands) (accuracy ± 5 mmHg) while the participant is in a seated position after 5 min of rest. The mean of the three systolic and diastolic BP measurements at 5 min intervals is calculated. BP values of 120/80 mmHg are considered normal references, and the ACC/AHA criteria are used for the diagnosis of arterial hypertension [151].

### 3.5. Blood Parameters

Blood samples are to be collected in the morning (approx. 8:00 am) by trained personnel at the Hospital University La Paz Extraction Unit coinciding with blood tests before chemotherapy to avoid more punctures and hospital visits than necessary. The complete methodology of the blood parameters, specialized biochemical analysis and saliva and stool microbiota are attached as supplementary material (File S3). 

#### 3.5.1. General Biochemical Analysis

General biochemical analysis are carried out in the Biochemistry Laboratory of the Hospital La Paz.

#### 3.5.2. Specialized Biochemical Analysis

Specialized biochemical analyses will be carried out in the Department of Biochemistry and Molecular Biology II University of Granada, Granada, Spain.

##### Plasma and Erythrocyte Collection

Venous blood samples are collected in EDTA-containing tubes and processed within the following 2 h.

##### Urine Collection

A sample of the morning urine is taken using a standardized procedure, aliquoted in 1 mL Eppendorf tubes, and immediately frozen at −80 °C until further analysis.

##### Fatty Acid Profile of Erythrocytes

Erythrocyte lipid extraction. For fatty acid methylation of erythrocytes, the procedure of Lepage & Roy (1988) [152] is followed.

Separation and quantification of fatty acids from erythrocyte lipids. Fatty acid methyl esters are identified and quantified by comparison of retention times with those of previously used authentic standards and confirmation by MS [153].

##### Evaluation of Corporal Oxidative Stress

Determination of 8-iso-PGF2α concentration in urine. The concentration of 8-iso-PGF2α in urine is determined by a highly specific and validated enzyme immunoassay (ELISA) as previously described [154].

Determination of 8-hydroxy-2′-deoxyguanosine concentration in urine. The concentration of 8-OHdG in urine is determined by a competitive enzyme-linked immunosorbent assay as reported elsewhere [154,155].

##### Evaluation of the Antioxidant Defense System

Plasma total antioxidant capacity (TAC). TAC is assessed using a spectrophotometric antioxidant assay kit (709001; Cayman). The ability of the antioxidants in the sample to prevent the oxidation of ABTS^®^ was compared to that of Trolox, a water-soluble tocopherol analog, and quantified as millimolar equivalents of Trolox [156].

Determination of oxidized and reduced glutathione in erythrocytes. A specific kit for the determination of glutathione by colorimetric means is used (Invitrogen, Waltham, MA, USA, catalog number: EIAGSHC (https://www.thermofisher.com/order/catalog/product/EIAGSHC (accessed on 1 October 2023)). 

Erythrocyte antioxidant enzyme activities. The hemoglobin (Hg) concentration in blood samples is determined with a colorimetric cyanmethemoglobin method. The activities of the antioxidant enzymes CAT, GR, GPx, and SOD are assayed by spectrophotometric-specific [157]. 

Determination of plasma antioxidants (retinol, β-carotene, and α-tocopherol). Plasma concentrations of retinol, β-carotene, and α-tocopherol are determined by solvent extraction and ultrahigh-pressure liquid chromatography coupled to mass spectrometry (UHPLC–MS) as reported elsewhere [158,159]. 

##### Plasma Cytokines

Based on previous reports [95,96,97,98], relevant molecules previously described as associated with inflammatory processes and cancer cachexia will be selected for their analysis in plasma. Namely, TNFα, IL-6, IL-1β, IFN-γ, IL-4, IL-10, IL-15, IL-1RA, IL-15, IL-1RA, sIL-6R, sTNFRI, sIL-6R, sTNFRI, sTNFRII, human leukemia inhibitory factor (LIF), human ciliary neurotrophic factor (CNTF) and human proteolysis-inducing factor/dermicidin (PIF/DCD) will be analyzed.

##### Plasma Metabolomics Analysis

A multiplex strategy (GlobalMet from EureCat) will be used for the separation and evaluation of both water-soluble and fat-soluble plasma metabolites (lipidomics) based on semitargeted analysis by GLC–MS (plasma metabolome analysis, nontargeted analysis by UPLC–MS and targeted analysis by UPLC–MS) [160,161,162].

### 3.6. Saliva and Stool Microbiota

#### 3.6.1. Saliva Sampling

The saliva samples obtained during the tests for taste and odor determination are poured into OMNIgene Oral OM-501 (DNA Genotek Inc., Ottawa, ON, Canada) tubes.

#### 3.6.2. Stool Sampling

Samples are collected directly by study participants at home using the OMNIgene Gut OM-200 (DNA Genotek Inc., Ottawa, ON, Canada) and stored immediately at −20 °C.

#### 3.6.3. Flow Cytometry

Microbial loads from saliva and fecal samples will be processed and analyzed by flow cytometry following a standardized procedure [163].

#### 3.6.4. Fecal DNA Amplification and Sequencing

Sequencing of bacterial DNA from both saliva and feces will be performed with a nanopore procedure (MinION™) that enables direct reading of long DNA sequences [164,165].

#### 3.6.5. Saliva and Stool Metagenomics

Potential functional profiles (metagenomic analysis) for the sequenced samples will be predicted using PICRUSt2 [166]. To infer the abundance of each gene family per sample, phylotype abundances will be corrected for their 16S rRNA gene copy number and then multiplied by their functional predictions [167].

## 4. Detailed Procedure

The clinical trial is divided into two phases (Selection and Intervention Phase), which include a selection visit (v0) and five more visits through the study (Figure 1).

### 4.1. Selection Phase

#### Visit 0 (v0, Selection Visit)

After clinical trial approval by the Ethics Committee, patients undergoing neoadjuvant treatment with chemotherapy or chemoradiotherapy for at least three months and malnutrition who were referred to the Clinical Nutrition and Dietetics Unit from the Oncology Service will be invited to participate in the study. If the patient is interested in participating, compliance with the study selection criteria is verified. When all requirements are met, an information-to-read document will be provided. Two researchers are in charge of recruiting patients, explaining the protocol, and providing the informed consent form. Once the patient’s doubts are clarified and if the patient is interested, informed consent will be signed.

During this visit, the following activities are carried out:

- Nutritional status assessment (GLIM Criteria);
- Electrogustometry;
- Taste strips test;
- Modified Taste and Smell Survey.

The following documentation is given to the patient to complete visit 1 (v1):

- Daily Food Record (3 days per holiday);
- Food Frequency Questionnaire;
- International Physical Activity Questionnaire;
- Quality of Life Questionnaire.

During this visit, the patient is informed about the next visit date and provided with the following:

- Blood sample extraction appointment;
- Stool container for microbiota and metagenome analysis;
- Urine container for 8-iso-PGF2α determination.

### 4.2. Experimental Phase

This phase includes 5 face-to-face visits. Except for visit 1, these visits are conditioned by the patients’ chemotherapy treatment sessions and are carried out in the following 4–7 days after their infusion, a period during which there is greater toxicity and taste affectation.

#### 4.2.1. Visit 1 (v1)

Once it is verified that patients meet the inclusion and exclusion criteria, the experimental phase’s first visit will be made. In this visit, the patient is randomized, and the following initial measures are taken:

- Health study (blood pressure and heart rate);
- Morphofunctional assessment:
  ▪ Anthropometric measurements;
  ▪ Electrical bioimpedance;
  ▪ Dynamometry;
  ▪ Nutritional ultrasound;
  ▪ Up and Go Test.
- Sniffin’ Stick Smell Test;
- Collection and measurement of saliva volume;
- Blood sample extraction coinciding with the prechemotherapy analysis:
  ▪ Biochemical parameters;
  ▪ Plasma metabolomic analysis;
  ▪ Plasma cytokine profile;
  ▪ Fatty acids from the erythrocyte membrane;
  ▪ Enzymatic antioxidant defense system in erythrocytes;
  ▪ Body oxidative stress in urine;
  ▪ Fecal microbiota and metagenome;
  ▪ Saliva microbiota and metagenome.

During this visit, the following documentation is collected:

- Daily Food Record (3 days per holiday);
- Food consumption frequency;
- International Physical Activity Questionnaire;
- Quality of life questionnaire;
- Feces sample;
- Urine sample.

Once the tests have been carried out, the patient will be instructed on the following:

- Nutritional treatment. If an oral nutritional supplement is needed, a polymeric, hypercaloric, and hyperproteic formula enriched in omega-3 fatty acids is prescribed depending on their energy requirements;
- Healthy eating guidelines for cancer patients using the book “Cooking with Science Against Cancer” as a support are given to the patient;
- Physical exercise guidelines.

During this visit, the study treatment (Miraculin-based food supplement or placebo) is delivered to the patient based on randomization, and the following documentation is delivered to bring the next visit (v2):

- Product efficacy satisfaction questionnaire (prechemotherapy);
- Next face-to-face visit date.

The following documentation will also be delivered to bring on the third visit (v3):

- Product consumption control daily sheet;
- Product consumption tolerance record sheet;
- Record sheet of adverse effects.

#### 4.2.2. Visit 2 (v2)

Carried out 4–7 days after the chemotherapy session, during this visit, the following measurements will be made:

- Anthropometric measurements;
- Electrogustometry;
- Smell and taste tests:
  ▪ Taste Strips Test;
  ▪ Sniffin ‘Sticks Smell Test.
- Collection and measurement of saliva volume;
- Taste and Smell Modified Survey;
- Product Efficacy Satisfaction Questionnaire (Post Chemotherapy).

The following behavioral reinforcements are also carried out on this visit:

- Nutritional treatment and physical activity;
- Consumption and registration of the assigned treatment;
- Tolerance and adverse effects registry.

At the end of this visit, the following documentation and material are given to the patient. These have to be delivered on the next visit (v3):

- Daily Food Record (3 days per holiday);
- International Physical Activity Questionnaire;
- Quality of Life Questionnaire;
- Blood sample extraction appointment;
- Feces container;
- Urine container.

#### 4.2.3. Visit 3 (v3)

This visit will take place ±1 month after visit 1 (v1) and 3–4 days after the patient’s chemotherapy treatment session. This visit is similar to visit 1. A detailed version of the performed assessments is included in the Supplementary Material (File S4).

#### 4.2.4. Visit 4 (v4)

This visit will take place ±2 months after visit 1 (v1) and 3–4 days after the patient’s chemotherapy treatment session. This visit is similar to visit 1. A detailed version of the assessments performed is included in the Supplementary Material (File S4). 

#### 4.2.5. Visit 5 (v5)

This visit will take place ±3 months after visit 1 (v1) and 3–4 days after the patient’s chemotherapy treatment session. This visit is similar to visit 1. A detailed version of the assessments performed is included in the Supplementary Material (File S4). 

## 5. General Characteristics of the Population

**Figure 5:**
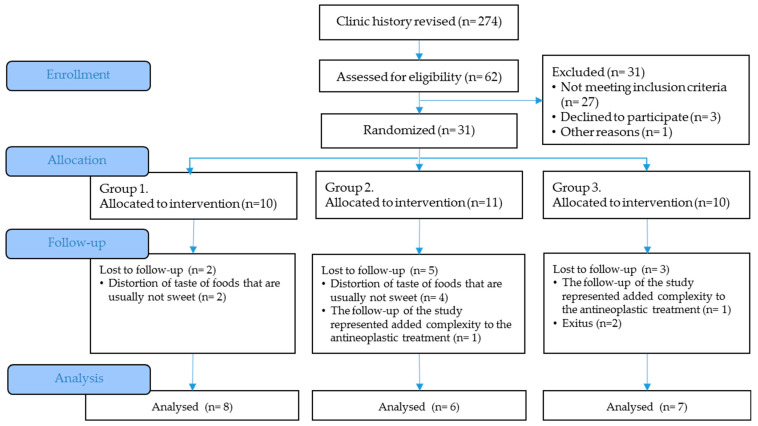
CONSORT Flow Diagram.

This section presents very preliminary results of the clinical trial. For the present study a total of 62 patients were evaluated for eligibility. Of them, 31 met the selection criteria and were randomized into three intervention groups (Figure 5). During follow-up, there were 10 dropouts, mainly due to the consumption of the supplement distorted the taste of some non-sweet acidic foods (n = 6) and because of the prescription added difficulty to their, already complex, antineoplastic treatment (n = 2). A final sample of 21 patients completed the clinical trial.

The sample was made up of 61.9% women and 38.1% men with a mean age of 59.8 ± 11.65 years old, all of them undergoing active treatment with chemotherapy and taste alteration measured by electrogustometry (Table 3). The average body mass index was 22.37 ± 3.67 kg/m^2^, classifying the patients as normal weight. However, the weight lost in the last 3 months was −7.60 ± 5.76%, with no significant differences between treatment groups. 

The most prevalent type of cancer was colorectal cancer (28.6%) followed by breast, lung, pancreas and liver cancers, each with 9.5%. Fasting glycemic control (105 ± 19 mg/dL) was within the normal reference parameters (74–106 mg/dL). The insulin concentration (12 ± 10 µU/mL) was normal in the entire population (3–25 µU/mL) as well as the lipid profile. All patients had systemic inflammation (CRP > 5 mg/dL). Both vitamins and minerals were found within the reference values except for vitamin D, which means blood concentration (21.25 ± 13.86 ng/mL) was insufficient (range 15–30 ng/mL).

## 6. Expected Results

To the best of the authors’ knowledge, this is the first clinical trial conducted to examine the effects of regular consumption of the miraculin-based food supplement, containing the miracle fruit extract DMB, on taste perception and nutritional status in malnourished cancer patients undergoing active antineoplastic treatment. Nonetheless, a double-blind, placebo-controlled, randomized controlled trial to evaluate a miracle berry supplement on taste alteration is also being carried out at the moment. However, this clinical trial is carried out exclusively in head and neck cancer treated with radiotherapy and subjectively assessing the disorder taste [168]. In our trial, we used a triple-blind randomized approach. The present study uses a freeze-dried extract from the fruits of *Synsepalum dulcificum*, approved as a novel food and considered safe for human consumption in Europe by EFSA [70]. Orally dissolving tablets rich in miraculin, a glycoprotein with the ability to change the taste perception of sour foods to a sweet taste [169], are used to improve taste perception in cancer patients.

Taste and smell disorders are common in patients with cancer in active antineoplastic treatment; taste disturbance prevalence ranges from approximately 20 to 86% [31] and persists throughout the treatment [32], while smell disorders are approximately 5 to 60% in cancer patients undergoing chemotherapy [4]. In this sense, cancer patients with taste and smell disorders develop food aversions that reduce food intake and promote poor nutrition, deteriorate quality of life [5,29] and increase the risk of malnutrition [27,28,170]. Poor nutritional status is related to an increase in complications and toxicity of treatment, interruption or failure of treatments, longer hospital stays, readmissions, and infections and is responsible for up to 10–20% of the mortality of oncologic patients [171].

This is the first study carried out with the novel miraculin-based novel food ingredient DMB, contained in the miraculin-based food supplement, that objectively assesses taste perception in cancer patients undergoing active antineoplastic treatment through electrogustometry. In this sense, electrical taste evaluation provides an accurate means for quantitatively assessing the taste system [24], and although electrical stimulation seems to activate the same brain regions as chemical stimulation [25] and has high test-retest reliability [172], to complete taste perception assessment in cancer patients, a chemical assessment of taste has been performed. Taste and smell senses are closely related to food taste perception [173]. Taking this into account, the orthonasal olfactory function of malnourished cancer patients in active antineoplastic treatment will also be evaluated in the present study.

The main expected effect is that malnourished cancer patients with taste distortion and consuming the miraculin-based food supplement improve their food taste perception and promote better food intake, improving malnutrition and associated risks.

Some studies have shown the effect of *Synsepalum dulcificum* fruit for treating taste disorders in cancer patients undergoing chemotherapy. A pilot clinical trial in cancer patients with dysgeusia revealed that the consumption was safe, patients showed a taste improvement and, when taste stabilization was considered, the improvement rate was higher [21]. Later, a pilot crossover clinical trial demonstrated improvements in taste disorders; some patients increased food intake, and some reported metallic taste disappearance [22]. Both studies, however, had a short duration, making it impossible to evaluate their impact on the nutritional status of the patients. Other drawbacks are their designs, which were unblinded, and the lack of validated tools to assess changes in taste. However, these pilot clinical trials have set a precedent for miracle fruit use in taste disorders in patients with dysgeusia due to antineoplastic treatment.

Malnutrition is highly prevalent in cancer patients, and up to 10–20% of cancer patients may die because of malnutrition rather than the tumor [43]. Therefore, its early identification is vital in early treatment. This clinical trial is based on the new criteria for the diagnosis of malnutrition, which includes the assessment of phenotypic criteria (nonvolitional weight loss, low BMI, reduced muscle mass) and etiological criteria (reduced food intake or assimilation, disease burden/inflammation presence) [23]. The diagnosis of malnutrition includes at least one phenotypic and one etiological criterion. Malnutrition severity is given by the phenotypic criteria presented, while the etiologic criteria are used to provide the context to guide the nutritional intervention and anticipated outcomes. In this sense, morphofunctional assessment has been used to support malnutrition diagnosis. This new assessment tool includes classic parameters (anthropometric parameters, biochemical parameters, food intake) and emerging parameters (bioelectrical impedance, nutritional ultrasound, dynamometry, functional test, biochemical parameters) [44] that allow an accurate approximation of the nutritional status of a patient. This evaluation includes functional assessments such as hand-grip strength or TUG used as a supportive measure of malnutrition diagnosis [23].

The parameters that the morphofunctional assessment incorporates are expected to improve after the regular consumption of the miraculin-based food supplement since a change in the patient’s nutritional status will have a positive impact on them.

Taste and smell disorders are common in patients undergoing antineoplastic treatments, reducing not only their food intake but also their quality of life [174,175]. Moreover, malnutrition by itself negatively impacts the quality of life and oncologic treatment. Sour and salty tastes seem to be the tastes that are most affected by antineoplastic treatments [176]. In this sense, it is expected that the regular consumption of the miraculin-based food supplement improves the acid food taste perception and helps to improve the quality of life of patients.

In addition to miraculin, miracle berry contains a large amount of antioxidants. Indeed, it is rich in terpenoids (74.4%), phenolic compounds (15.8%), and flavonoids (11.9%) and exhibits a high antioxidant activity (18.9%) [177]. It also has anticancer abilities due to the different amides existing in the miracle fruit [178]. In cancer, high oxidative stress and profound alterations of the antioxidant defense system occur [101,102,103,104], partly associated with the inflammatory state [102] and partly related to the altered nutritional status affecting key molecules in the maintenance of oxidative homeostasis such as retinol, beta-carotene, vitamin E, vitamin C, increased ratio of oxidized to reduced glutathione, etc. [100,103,113,114]. Consequently, it is expected that the administration of DMB will contribute to decreasing the degree of oxidative stress, as evaluated in the present study through the urinary excretion of the biomarkers F2-alpha-isoprostanes and 8-hydroxy-deoxy-deoxy-guanosine [154,155,179,180]. Likewise, an improvement in both nonenzymatic and enzymatic antioxidant defense status [115] is expected, mainly based on the improvement in food intake and nutritional status of the treated patients.

Inflammation is a constant in cancer. As previously indicated, in addition to the production of proinflammatory cytokines and acute phase reactant proteins, tumor cells produce specific cytokines such as LIF and PIF, among others [95,96,97,98]. In the present project, it is expected that the improvement in nutritional status will contribute decisively to the reduction in the inflammatory process associated with cancer and, therefore, to a reduction in both proinflammatory cytokines and cancer-induced secretion factors themselves. Likewise, an increase in anti-inflammatory cytokines such as IL-4 and IL-10, which are decreased in tumor processes [95,96], would presumably occur.

As has been indicated before, malnutrition usually affects cancer patients. Beyond changes in the plasma proteome, this state of malnutrition causes changes in numerous low molecular weight biomarkers, many of which are derived from cellular metabolic activity, such as intermediary metabolism compounds, amino acids and amino acid derivatives, and compounds largely derived from nutrients and bioactive compounds derived from food intake [116,117]. The improvement of the latter and nutritional status should be associated with a metabolome closer to that of the healthy subject. In the present project, the study of the metabolome of compounds of molecular weight lower than 500 Da, as well as the lipidomic analysis, especially of lipid species (mono acyl, diacyl, and triacylglycerols, phospholipids, acylcarnitine derivatives, and oxylipins) [160,161,162], will allow us to know to what extent the miraculin supplementation translates into the improvement of the plasma metabolome in cancer subjects through the improvement of the nutritional status of the patients. On the other hand, it can be hypothesized that a higher and better quality food intake will be translated into higher intakes of essential fatty acids that should result in an improvement in the status of long-chain polyunsaturated fatty acids [91,92,93]. In this work, we plan to determine the status of essential fatty acids and their polyunsaturated derivatives by determining the fatty acid profile of the erythrocyte membrane [153]; given that supplementation with miraculin is extended for 12 weeks, this time is sufficient for the total pool of erythrocytes to be completely renewed, and therefore any change in the fatty acid profile would be indicative of improved nutritional status.

Numerous studies carried out in recent years using metagenomic techniques indicate that diet is a powerful agent involved in the establishment of specific microbiota, both in the oral cavity and in the colon [167]. In the present study, we addressed changes in the saliva and stool microbiota in cancer patients. Treatment with DMB and the increase in salivation related to some of its active compounds, such as some amides, as well as the change induced in food intake as a consequence of the improvement of taste and smell alterations, should result in relevant changes both in the salivary microbiome, which should be closer to that of the healthy subject, and in the bacterial pattern of the feces, indicative of the microbiome of the distal colon. Therefore, an improvement in the dysbiosis observed in both saliva and intestine [120,121,122,123,124,125,126,127,128,129] is expected in patients supplemented with miraculin.

One disadvantage of clinical trials is the potential decrease in the number of patients, which may be further reduced due to dropouts throughout the study. However, the primary objective of the pilot clinical trial is to comprehensively assess various parameters affected in cancer patients, who are susceptible to improvement through regular consumption of the miraculin-based food supplement.

The outcomes of this pilot clinical trial are anticipated to demonstrate whether the regular consumption of a miraculin-based food supplement, whose active principle is the miracle berry extract DMB, has a positive effect on taste distortion and the nutritional status of malnourished cancer patients undergoing active antineoplastic treatment and to determine the dosage at which maximum benefits are achieved.

## Figures and Tables

**Table 1:** Nutritional composition of the miraculin-based food supplement and placebo.

		150 mg DMB + 150 mg Freeze-Dried Strawberry	300 mg DMB	Placebo (300 mg Freeze-Dried Strawberry)
Energy	kcal	0.99	1	0.97
Carbohydrates	mg	194	234	154
Sugars	mg	156	162	150
Fiber	mg	26	6	46
Proteins	mg	20	15	24
Lipids	mg	9	5	12
Saturated fatty acids	mg	2	2	1
Sodium chloride	mg	0.1	0.1	0.03
Humidity	mg	4	4	5
Ash	mg	12	14	15
Miraculin	mg	2.8	5.5	0

Nutritional composition provided by Medicinal Gardens, S.L.

**Table 2:** GLIM Criteria, phenotypic and etiologic criteria for the diagnosis of malnutrition.

	Phenotypic Criteria			Etiologic Criteria	
	Weight Loss (%)	Low BMI (kg/m^2^)	Reduced Muscle Mass	Reduced Food Intake or Assimilation	Inflammation
**Moderate Malnutrition**	>5% within past 6 months or >10% beyond 6 months	<20 if <70 years, or <22 if ≥70 years	Reduced by validated body composition measuring techniques	≤50% of ER > 1 week, or any reduction for > 2 weeks or any chronic gastrointestinal condition that adversely impacts food assimilation or absorption	Acute disease/injury or chronic disease-related
**Severe** **Malnutrition**	>10% within past 6 months or >20% beyond 6 months	<18.5 if <70 years, or <20 if ≥70 years	Severe deficit		

BMI, body mass index; ASMI, Appendicular Skeletal Muscle Index; ER, energy requirements.

**Table 3:** General characteristics of the population (X ± SD).

		Group 1	Group 2	Group 3	*p*-Value
Sex (women)	(%)	62.5	33.3	85.7	0.153
Age	(years)	60.13 ± 16.23	58.83 ± 4.75	60.14 ± 11.19	0.976
BMI	(kg/m^2^)	20.79 ± 3.05	23.13 ± 3.83	23.53 ± 4.05	0.310
Type of cancer					
Head and neck	(%)	0.00	16.70	0.00	0.796
Colorectal	(%)	25.00	33.30	28.60	
Esophagus	(%)	12.50	0.00	14.30	
Stomach	(%)	0.00	0.00	14.30	
Liver	(%)	0.00	16.70	14.30	
Breast	(%)	0.00	16.70	14.30	
Neuroendocrine	(%)	12.50	0.00	0.00	
Ovary	(%)	12.50	16.70	0.00	
Pancreas	(%)	12.50	0.00	0.00	
Lung	(%)	12.50	0.00	14.30	
Others	(%)	12.50	0.00	0.00	
Chemotherapy	(%)	100.0	100.0	100.0	-
Radiotherapy	(%)	25.00	20.00	0.00	0.657
Weight lost	(%)	8 ± 17.16	−8.23 ± 3.12	−6.61 ± 6.98	0.867
Electrogustometry	(dB)	17.16 ± 12.46	20.5 ± 15.88	19.29 ± 15.71	0.909
Glucose	(mg/dL)	105 ± 17	97 ± 12	109 ± 26	0.578
Insulin	(µU/mL)	10 ± 11	13 ± 9	13 ± 9	0.811
Total Cholesterol	(mg/dL)	177 ± 29	182 ± 29	192 ± 25	0.589
HDL	(mg/dL)	54 ± 22	48 ± 18	59 ± 26	0.664
LDL	(mg/dL)	103 ± 26	108 ± 33	106 ± 21	0.946
Triglycerides	(mg/dL)	108 ± 67	139 ± 64	130 ± 35	0.588
CRP	(mg/dL)	5 ± 8	7 ± 4	7 ± 8	0.790
Ferritin	(ng/mL)	158 ± 129	175 ± 156	201 ± 149	0.849
Vitamin B12	(pg/mL)	730 ± 594	733 ± 653	765 ± 571	0.993
Serum folate	(ng/mL)	15 ± 7	14 ± 6	17 ± 7	0.674
Vitamin D	(ng/mL)	24 ± 10	19 ± 17	18 ± 15	0.695
Vitamin A	(µg/mL)	0.61 ± 0.25	0.72 ± 0.23	0.52 ± 0.09	0.244
Vitamin E	(µg/mL)	13 ± 3	16 ± 3	15 ± 2	0.337
Selenium	(µg/L)	75 ± 16	73 ± 13	70. ± 11	0.817
Zinc	(mg/dL)	1179 ± 223	1152 ± 171	1171 ± 325	0.982

BMI, body mass index; CRP, C-reactive protein.

## Data Availability

This article contains the detailed description of the CLINMIR study protocol. Analysis of saliva, plasma and fecal samples are currently being carried out. The analytical set of data generated during the study will be published and communicated to Nutrients.

## Supplementary Materials

The following supporting information can be downloaded at: https://www.mdpi.com/article/10.3390/nu15214639/s1, File S1. SPIRIT checklist; File S2. Informed consent form; File S3. Materials and Equipment; File S4. Detailed Procedure. References [181,182,183,184,185] are cited in the supplementary materials.
