# Supplementary material for: Effect of Regular Consumption of a Miraculin-Based Food Supplement on Taste Perception and Nutritional Status in Malnourished Cancer Patients: A Triple-Blind, Randomized, Placebo-Controlled Clinical Trial-CLINMIR Pilot Protocol
Source: Nutrients. 2023 Nov 1;15(21):4639. doi: 10.3390/nu15214639 (PMC10648678; doi:10.3390/nu15214639)
Supplement: Supplementary file 1 [file nutrients-15-04639-s001.zip › File S2. Informed consent form.pdf]

File S2. Informed consent form

EFFECT OF HABITUAL CONSUMPTION OF TASTECARE [DRIED MIRACLE BERRY (DMB)] ON THE OLFACTORY-GUSTATIVE PERCEPTION AND NUTRITIONAL STATUS OF CANCER PATIENTS UNDERGOING CHEMOTHERAPY AND/OR RADIOTHERAPY TREATMENT AND MALNUTRITION

Promoter: Medicinal Gardens S.L.

1. What is it and what does this study pursue?

The Nutrition Department from La Paz University Hospital in Madrid will carry out a research study in which we invite you to participate. The study consists of evaluating the effect of habitual consumption of a suckable and dispersable tablet rich in miraculin on the perception of taste and smell and the nutritional status of cancer patients undergoing chemotherapy and/or radiotherapy treatment and malnutrition.

Miraculin is a protein present in the dried berries of *Synsepalum dulcificum* (DMB®, dried miracle berries). Miraculin has the property of changing the perception of sour flavors and, to a lesser extent, bitter flavors into sweet flavors. In this sense, one of the side effects of chemotherapy and/or radiotherapy treatment in cancer patients is the alteration of taste and smell perception. The presence of these alterations results in patients reporting a bad taste in the mouth, taste distortion, and greater sensitivity to odors. The presence of taste and smell alterations causes patients to modify their diet, which often results in reduced energy and nutrient intake, a greater probability of weight loss and a negative impact on nutritional status and quality of life.

In this sense, it is possible that the consumption before each main meal (breakfast, lunch and dinner) of a chewable tablet of DMB®, rich in miraculin, can improve the sensory perception of the food consumed, improving intake and, potentially, their nutritional status. For this reason, a clinical trial has been proposed to evaluate the effect of the consumption of DMB®, rich in miraculin, on the perception of smell and taste and the nutritional status of cancer patients undergoing chemotherapy and/or radiotherapy who present malnutrition.

Before you decide whether or not to participate, you should understand the reason for the research and what it involves. You must read this document carefully and ask the person in charge or someone from the research project team any questions that may arise.

2. How is the study done?

2.1 Treatment administered

The treatment will consist of consuming a chewable tablet of DMB® or placebo just before each main meal (breakfast, lunch and dinner) for 3 months. During this period of time, you will be evaluated in five face-to-face visits at the beginning (v1), one week later (v2), a month (v3), two months (v4) and 3 months (v5) in addition to the selection (vs).

It is very important that you consume exactly the amount of chewable tablet we ask for. You should do it as if it were a medicine since, as such, a smaller intake may not produce the desired effect or, on the contrary, it may not be beneficial if it is taken in larger quantities. If you cannot take the full amount prescribed, you will notify your doctor.

2.2 Methodology used

If you decide to participate in this study, you should know that the total time to carry it out will be 3 months.

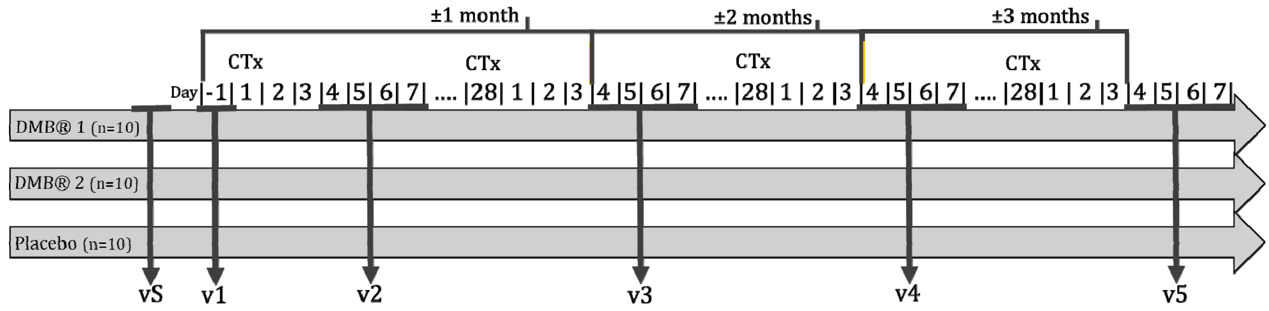

First, the researcher will assess whether you meet the study selection criteria. This will be done by applying criteria for the diagnosis of malnutrition (GLIM Criteria) and a survey on taste and smell that consists of 12 questions. To assess whether the perception of taste and smell has been altered, electrogustometry (a painless test in which the threshold of taste detection is assessed through an electrical pulse) and a test with taste strips will be performed. If so, the study will be explained to you, and the doctor or researcher will answer your questions about it. If it is confirmed that you meet the selection criteria to participate in the study, you may do so after signing the informed consent form.

During the screening visit, you will be provided with a flyer for the analysis, a feces and urine container for their corresponding analyses, and three questionnaires to determine the type of diet you carry out, what your physical activity is and how the disease and its treatment affect your health and quality of life.

Visit 1 (v1, before chemotherapy):

Before your visit to the Nutrition Office, you will have to go with your analytics flyer to the blood collection area where a blood sample will be collected by venipuncture. Once in the Nutrition Office, the questionnaires delivered in the screening visit will be collected, and the stool and urine samples will be refrigerated.

During this visit, you will be randomly assigned to one of three study groups. In this way, you could be part of the group that consumes the DMB® (in one of its two doses) or the control group that consumes a placebo.

During this visit, we will perform the following tests:

- Health study (blood pressure and heart rate)
- Morphofunctional assessment:
  - Anthropometric measurements (weight, height, and circumference)
  - Electrical bioimpedance (painless test to know your body composition)
  - Dynamometry (hand grip strength)
  - Nutritional ultrasound (painless test to assess muscle quantity)
  - Up and Go Test (timing getting up from a chair, walking 3 meters and coming back)
- Sniffin'stick test (Smell test)
- Collection and measurement of saliva volume

During this visit, nutritional treatment and the guidelines for healthy eating and physical exercise will be prescribed. You will also be given the assigned treatment and the consumption indication. To keep

a record of the consumption of the treatment, you will be provided with a diary sheet where you must write down your consumption or if you have forgotten anything. After the first consumption of the treatment, you must complete a satisfaction questionnaire on the efficacy of the product (prior to your chemotherapy treatment). During this visit, you will be given a document to record the tolerance for consumption of the product and the record of possible adverse effects.

Finally, the research staff will indicate the date for the next face-to-face visit (v2).

The approximate time of this visit will be 45 minutes.

Visit 2 (**v2**, after 3-4 days of chemotherapy):

During this visit, the satisfaction questionnaire on the efficacy of the product completed prior to chemotherapy will be collected. During this visit, the following tests will be performed:

- Anthropometric measurements (weight)
- Electrogustometry (painless test in which the threshold of taste detection is assessed through an electrical pulse)
- Olfactory-gustatory tests:
  - Test Strips (taste test with taste strips)
  - Sniffin' Sticks Smell Test (smell test)
- Saliva volume
- Taste and smell survey
- Product efficacy satisfaction questionnaire (postchemotherapy)

During this visit, you will be provided with a document to record your food consumption for three days (one of which will be a holiday), a physical activity questionnaire, and your quality of life. In addition, you will receive reinforcements on nutritional treatment and physical activity, consumption and registration of the treatment and on the registration of tolerance and adverse effects.

Finally, during this visit, you will be assigned a date to carry out the next face-to-face visit (v3), your analytical flyer, and the containers for the stool and urine samples that you must bring with you to the next visit.

The approximate time of this visit will be 50 minutes.

Visit 3 (**v3**, after ±1 month from v1, 4-5 days after chemotherapy):

Before your visit to the Nutrition Office, you will have to go with your analytics flyer to the blood collection area where a blood sample will be collected by venipuncture. Once in the Nutrition Office, the questionnaires delivered in the previous visit will be collected, and the stool and urine samples will be refrigerated.

During this visit, the following actions will be carried out:

- Nutritional status assessment
- Blood pressure and heart rate
- Morphofunctional assessment:
  - Anthropometric measurements (weight, height, and circumference)
  - Electrical bioimpedance (painless test to know your body composition)
  - Dynamometry (hand grip strength)
  - Nutritional ultrasound (painless test to assess muscle quantity)
  - Up and Go Test (timing getting up from a chair, walking 3 meters and coming back)

- Electrogustometry (painless test in which the threshold of taste detection is assessed through an electrical pulse)
- Olfactory-gustatory tests:
  - Test Strips (taste test with taste strips)
  - Sniffin' Sticks Smell Test (smell test)
- Saliva volume
- Taste and smell survey
- Product efficacy satisfaction questionnaire

At this visit, you will be given a new batch of study treatments, and any leftovers will be collected. It is important that you also bring empty containers with you. During this visit, you will also be given a document to register your food consumption in three days (one of which will be a holiday), a physical activity questionnaire, and a quality of life questionnaire. To monitor the consumption of the treatment, you will be provided with a diary sheet to record it until your next visit. In addition, you will be given a record sheet of product consumption tolerance and adverse effects.

During this visit, the nutritional and physical activity treatment, the consumption and recording of the treatment, and the recording of tolerance and adverse effects will be reinforced. You will also be given the date for the next face-to-face visit (v4) as well as your analytical flyer.

The approximate time of this visit will be 60 minutes.

Visit 4 (v4, after ±2 months from v1, 4-5 days after chemotherapy):
Before your visit to the Nutrition Office, you will have to go with your analytics flyer to the blood collection area where a blood sample will be collected by venipuncture. Once in the Nutrition Office, the questionnaires delivered in the previous visit will be collected.
During this visit, the following actions will be carried out:

- Nutritional status assessment
- Blood pressure and heart rate
- Morphofunctional assessment:
  - Anthropometric measurements (weight, height, and circumference)
  - Electrical bioimpedance (painless test to know your body composition)
  - Dynamometry (hand grip strength)
  - Nutritional ultrasound (painless test to assess muscle quantity)
  - Up and Go Test (timing getting up from a chair, walking 3 meters and coming back)
- Electrogustometry (painless test in which the threshold of taste detection is assessed through an electrical pulse)
- Olfactory-gustatory tests:
  - Test Strips (taste test with taste strips)
  - Sniffin' Sticks Smell Test (smell test)
- Saliva volume
- Taste and smell survey
- Product efficacy satisfaction questionnaire

At this visit, you will be given a new batch of study treatments, and any leftovers will be collected. It is important that you also bring empty containers with you. During this visit, you will also be given a

document to register your food consumption on three days (one of which will be a holiday), a frequency of food consumption in the last three months, a physical activity questionnaire and a quality of life questionnaire. To monitor the consumption of the treatment, you will be provided with a diary sheet to record it until your next visit. In addition, you will be given a record sheet of product consumption tolerance and adverse effects.

During this visit, nutritional treatment and physical activity will be reinforced. You will also be given the date for the next face-to-face visit (v5) as well as your analytical flyer, a container for stool and urine samples that you must bring with you.

The approximate time of this visit will be 50 minutes.

Visit 5 (v5, after ±3 months of v1, 4-5 days after chemotherapy):
Before your visit to the Nutrition Office, you will have to go with your analytics flyer to the blood collection area where a blood sample will be collected by venipuncture. Once in the Nutrition Office, the questionnaires delivered in the previous visit as well as the stool and urine samples will be refrigerated.

During this visit, the following actions will be carried out:

- Nutritional status assessment
- Blood pressure and heart rate
- Morphofunctional assessment:
  - Anthropometric measurements (weight, height, and circumference)
  - Electrical bioimpedance (painless test to know your body composition)
  - Dynamometry (hand grip strength)
  - Nutritional ultrasound (painless test to assess muscle quantity)
  - Up and Go Test (timing getting up from a chair, walking 3 meters and coming back)
- Electrogustometry (painless test in which the threshold of taste detection is assessed through an electrical pulse)
- Olfactory-gustatory tests:
  - Test Strips (taste test with taste strips)
  - Sniffin' Sticks Smell Test (smell test)
- Saliva volume
- Taste and smell survey
- Product efficacy satisfaction questionnaire

In this final visit, nutritional treatment and physical activity will be reinforced.

The approximate time of this visit will be 50 minutes.

3. What are the expected benefits and potential risks of this study?

3.1 Benefits

It is expected that, since miraculin has the ability to change sour and, to a lesser extent, bitter tastes to sweet, the habitual consumption of DMB® improves the olfactory-gustatory perception of food and potentially the nutritional status of the malnourished patient. However, you should know that it is

also possible not to obtain the expected results. In this sense, there is no guarantee that you will obtain a direct benefit as a result of your participation in the study.

3.2 Risks

The study products have undergone quality controls and meet all the criteria and requirements necessary to be consumed. The DMB® supplement has been approved as a novel food (novel food) in accordance with Regulation (EU) 2015/2283 and upon request to the European Commission through the EFSA Panel on Nutrition, Novel Foods and Food Allergens. The positive report by EFSA establishes that the DMB® product is safe for human consumption. The consumption of DMB® does not represent any risk to your health.

The tests to which you will be subjected throughout the study will not pose any risk to your health.

4. Your participation is voluntary

If you wish to participate in this study, you must notify your doctor. Your participation is voluntary. You should know that you can decide to stop participating at any time by telling your doctor without having to give any reason. In this case, you will be asked if your decision is related to any adverse event from the consumption of the food supplement. Whether you do not want to participate or drop out of the study, you will be treated according to standard clinical practice.

If during the course of the study any relevant information or data arise of your interest or that may condition your participation in the study, it will be communicated to you by the principal researcher.

Your doctor may also withdraw you from this study if he deems it appropriate; also for not going to the scheduled visits or for not consuming the supplement as indicated.

5. Review of Original Documents, Confidentiality and Personal Data Protection

5.1 Confidentiality and document review

You understand and agree that to guarantee the reliability of the data collected in this study, it will be necessary for representatives of the health authorities and/or members of the Clinical Research Ethics Committee to have access to your clinical history, committing to the strictest confidentiality.

The processing of your personal data will always be in accordance with Ley Orgánica 3/2018, of December 5, on the Protection of Personal Data and guarantee of digital rights and Regulation (EU) 2016/679 of the European Parliament and of the Council of April 27, 2016 on the protection of individuals with regard to the processing of personal data and the free circulation of these data. The study participant may exercise their rights of access, rectification, deletion, position, limitation of treatment and portability, to the extent that they are applicable, through written communication to the Data Controller, with address at Hospital Universitario La Paz, Paseo de la Castellana 261, 28046 Madrid, specifying your request, along with your ID or equivalent document. Likewise, you will have the possibility of filing a claim with the Spanish Agency for Data Protection (Agencia Española de Protección de Datos)

The personal data that are required (for example, age, sex, health data) are those necessary to cover the objectives of the study. Your name will not appear in any of the study reports, and your identity will not be revealed to anyone except to comply with the purposes of the study and in case of medical

emergency or legal requirement. Any personal information that may be identifiable will be kept and processed under secure conditions with the purpose of determining the results of the study. These may be communicated to the health authorities and, eventually, to the scientific community through congresses and/or publications and scientific dissemination seminars/conferences.

The researchers have the express commitment to issue a report with the results of the study regardless of their meaning or medical or commercial relevance, which will be delivered both to the promoter of the research study and to the Ethics Committee.

6. Information you should know

6.1 Economic compensation

Study participants will not receive financial compensation for participation in the study.

6.2 Additional Information

In the event of any eventuality that may arise while you are participating in this study or for any questions about it that you may have after reading this document, please contact:

Study name	Effect of habitual consumption of dried miracle berry (dmb) on the olfactory-gustative perception and nutritional status of cancer patients undergoing chemotherapy and/or radiotherapy treatment and malnutrition
Principal investigator	Dra. Palma Milla
Adress	Nutrition Department. Hospital Universitario La Paz (Madrid)
Phone	+34 xxx xxx xxx

A copy of this signed and dated informed consent form will be delivered.

CONFIDENTIALITY AND DATA PROTECTION

CONSENT FOR RESEARCH STUDIES

Through this letter and in compliance with current regulations on data protection, I am informed and expressly consent to the processing of the data from my medical history as well as those resulting from their participation in *the effect of habitual consumption of dried miracle berry (dmb) on the olfactory-gustative perception and nutritional status of cancer patients undergoing chemotherapy and/or radiotherapy treatment and malnutrition* study. The data controller is Hospital Universitario La Paz (including Hospital Carlos III-Hospital Cantoblanco), whose Data Protection Delegate (DPD) is the "PDP Committee of the Department of Health of the Community of Madrid" with address in Plaza Carlos Trías Bertrán n°7 (Edificio Soluble) Madrid 28020 (protecciondedatos.sanidad@madrid.org). The purpose of the study is to evaluate the effect of olfactory-gustatory perception and the nutritional status of cancer patients undergoing treatment with chemotherapy and/or radiotherapy who present malnutrition.

The legal basis that legitimizes the treatment is your consent, as well as Ley 14/2007, of July 3, on Biomedical Research and other current legislation on the matter. For this purpose, your data will be kept for the years necessary to comply with the obligations stipulated in the applicable current regulations, as well as long as it is useful for the purpose for which it was obtained, and in any case, for at least five years. Access to your personal information will be restricted to the study doctor(s), their collaborators and other personnel participating in the study, health authorities, the Hospital's Research Ethics Committee and the promoter's monitors and auditors, who will be subject to the duty of secret inherent to their profession, when needed, to verify the study data and procedures but always maintain their confidentiality in accordance with current legislation. No additional data communications will be made, except in those cases required by law.

By providing your data, you guarantee that you have read and expressly accepted their treatment as indicated. You may exercise your rights of access, rectification, deletion, opposition, limitation of treatment and portability, to the extent that they are applicable, through written communication to the data controller, with address at Hospital Universitario La Paz, Paseo de la Castellana 261, 28046 Madrid, specifying your request, together with your ID or equivalent document. Likewise, we inform you of the possibility of filing a claim with the Spanish Data Protection Agency (C/Jorge Juan, 6 Madrid 28001) (www.agpd.es).

And for the record, I signed it in Madrid on _____ of ________________ 20___.

SIGNED:

Mr./Mrs. ____, with N.I.F. _________________________, in my own name and representation, or in the name and representation of Mr./Mrs. _____________________________________, with N.I.F. _____________________________ (such condition must be proven).
