# Supplementary material for: Effect of Regular Consumption of a Miraculin-Based Food Supplement on Taste Perception and Nutritional Status in Malnourished Cancer Patients: A Triple-Blind, Randomized, Placebo-Controlled Clinical Trial-CLINMIR Pilot Protocol
Source: Nutrients. 2023 Nov 1;15(21):4639. doi: 10.3390/nu15214639 (PMC10648678; doi:10.3390/nu15214639)
Supplement: Supplementary file 1 [file nutrients-15-04639-s001.zip › File S3. Materials and Equipement.pdf]

nutrients

*File S3. Materials and Equipment*

# Effect of Regular Consumption of a Miraculin-Based Food Supplement on Taste Perception and Nutritional Status in Malnourished Cancer Patients: A Triple-Blind, Randomized, Placebo-Controlled Clinical Trial−CLINMIR Pilot Protocol

## 3. Materials and Equipment

### 3.1 *Blood Parameters*

Blood samples were collected in the morning (approx. 8:00 am) by trained personnel at the Hospital University La Paz Extraction Unit coinciding with blood tests before chemotherapy to avoid more punctures and hospital visits than necessary. Fasting samples were collected in vacuum tubes, labeled, transported, and then centrifuged for 10 min at 1500×g for general biochemical analysis. Samples for specialized analysis were prepared as indicated below. Sample aliquots were prepared and labeled according to a numerical code and properly stored at -20 °C or -80 °C as appropriate.

#### 3.5.1 General biochemical analysis.

In addition to the assessment of the hemogram and coagulation test, the biochemical parameters to be assessed at each of the visits are taken from the patient's routine analysis closest to the study visit (v1, v3, v4, v5). The following parameters were analyzed: albumin, prealbumin, C-reactive protein (ultrasensitive), glucose, lipid profile (total cholesterol, triacylglycerols, HDL-c, LDL-c), transaminase GOT and GPT activities, uric acid, creatinine, fat-soluble vitamins (A, D, E), other vitamins (B9 (folates) and B12), and minerals (iron, zinc and selenium).

All these analyses were carried out in the Biochemistry Laboratory of the Hospital La Paz using methods developed by the researchers of this institution with an Olympus AU5400 Automated Chemistry Analyzer (Olympus Corporation, Izasa, CA, USA) (albumin, prealbumin, RBP, glucose, lipid profile (TC, TG, HDL, LDL), safety parameters (transaminases, urate, creatinine), fat-soluble vitamins (A, D, E), Vitamin B12, folate, iron metabolism, Zn, Se. Hemogram, lymphocytes, coagulation, PCRus).

#### 3.5.2 Specialized biochemical analysis

##### 3.5.2.1 Plasma and erythrocyte collection

Venous blood samples were collected in EDTA-containing tubes and processed within the following 2 hours. After centrifugation at 3500 × g for 10 min, the plasma was divided

into aliquots for specialized biochemical analyses of plasma cytokines, nonenzymatic antioxidant defense system analytes and metabolomics and frozen at -80 °C until transport and analysis. The cell pellet was immediately washed three times with 0.9% NaCl isotonic solution, and the packed erythrocytes were collected and stored at -80 °C until analysis for the red blood cell enzymatic-antioxidant defense system and for lipid extraction and analysis. Erythrocyte fatty acid profile.

3.5.2.2 Urine collection

A sample of the morning urine will be taken using a standardized procedure in visits 1 and 5; samples will be aliquoted in 1 ml Eppendorf tubes and immediately frozen at -80 °C until further analysis.

3.5.2.3 Fatty acid profile of erythrocytes

*Erythrocyte lipid extraction*

For the extraction of erythrocyte lipids, approximately 1 mL of washed cells was successively treated with 3 mL of isopropanol containing 50 mg/L butylated hydroxytoluene as an antioxidant, 2 mL of isopropanol and 2 mL of hexane. After centrifugation for 10 min at 3000 g at 4 °C, the upper hexane phase was collected and re-extracted three times with 2 mL of hexane. The hexane extracts were combined and dried under nitrogen. For fatty acid methylation of erythrocytes, the procedure of Lepage & Roy (1988) [1] was followed. Briefly, a direct methylation procedure was carried out in 5.0 mL of methanol-acetyl chloride 50:1 (v/v). To stop the reaction, 3 ml of 6.0% $K_2CO_3$ was added. After adding 150 μL of hexane, shaking and centrifuging, the upper phase was separated and dried under nitrogen.

*Separation and quantification of fatty acids from erythrocyte lipids*.

Methylated fatty acids were resuspended in 100 μL of hexane, and 1 μL was injected into a gas–liquid chromatograph coupled to mass spectrometry (GLC–MS) with a capillary column (60 m x 32 mm inner diameter; 20 μm film thickness) impregnated with SP2330 FS (Supelco, Bellefonte, CA, USA). Fatty acid methyl esters are identified and quantified by comparison of retention times with those of previously used authentic standards and confirmation by MS [2].

3.5.2.4 Evaluation of corporal oxidative stress

*Determination of 8-iso-PGF2α concentration in urine*

The concentration of 8-iso-PGF2α in urine was determined by a highly specific and validated enzyme immunoassay (ELISA) as previously described [3] (EA85 Oxford

Biomedical Research, Oxford, MI, USA) (CV: 14.13%). The detection limit of the analysis was 23 pmol/L.

*Determination of 8-hydroxy-2′-deoxyguanosine concentration in urine*

The concentration of 8-OHdG in urine was determined by a competitive enzyme-linked immunosorbent assay (ELISA) kit (JAICA, Fukuroi, Japan) (CV: 5.73%) as reported elsewhere [3,4]. Briefly, the primary monoclonal antibody and the sample or standard are added to microplates previously coated with 8-OHdG. After incubation for one hour at 37 °C, the plates were washed with phosphate-buffered saline (PBS). Horseradish peroxidase-conjugated secondary antibody was then added to each well, incubated at 37 °C for another hour and washed again with PBS. After the addition of the enzyme substrate to each well, the plate was allowed to react at room temperature for an additional 15 min. Finally, the reaction was terminated with phosphoric acid. The absorbance of each well was determined at 450 nm with a microplate reader. The determination range was 0.125-10 ng/mL. The concentration of 8-OHdG was adjusted according to urine levels of creatinine and is expressed as ng 8-OHdG/mg creatinine [5,6].

3.5.2.5 Evaluation of the antioxidant defense system

*Plasma total antioxidant capacity (TAC)*

TAC was assessed using a spectrophotometric antioxidant assay kit (709001; Cayman). The antioxidant assay can be used to measure the total antioxidant capacity of plasma, serum, urine, saliva, or cell lysates. Water- and lipid-soluble antioxidants are not separated in this protocol, so the combined antioxidant activities of all their constituents, including vitamins, proteins, lipids, glutathione, uric acid, etc., are evaluated. The assay is based on the ability of the antioxidants in the sample to inhibit the oxidation of ABTS® (2,2'-azino-di-[3-ethylbenzozoline sulfonate]) to ABTS® by metamyoglobin. The amount of ABTS® produced can be monitored by reading the absorbance at 750 nm or 405 nm. Under the reaction conditions used, the antioxidants in the sample cause suppression of the absorbance at 750 nm or 405 nm to a degree that is proportional to their concentration.2-5 The ability of the antioxidants in the sample to prevent the oxidation of ABTS® was compared to that of Trolox, a water-soluble tocopherol analog, and quantified as millimolar equivalents of Trolox [7].

*Determination of oxidized and reduced glutathione in erythrocytes*

A specific kit for the determination of glutathione by colorimetric means was used (Invitrogen, catalog number: EIAGSHC (https://www.thermofisher.com/order/catalog/product/EIAGSHC). This kit is designed to

quantitatively measure reduced glutathione (GSH) and oxidized glutathione (GSSG) present in a variety of samples, including erythrocytes. No separation or washing is needed. A GSSG standard was provided to generate a standard curve for the assay, and all samples were read on the standard curve. The kit uses a colorimetric substrate that reacts with the free thiol group of GSH to produce a highly colored product. The reagents supplied are in solution, so a simple dilution should be carried out for use in the assay. If 2-vinylpyridine is used to block any free GSH in the sample, oxidized glutathione (GSSG) can be determined. Samples that have not been treated with 2-vinylpyridine allow the determination of total GSH levels. The concentration of free GSH in the sample was calculated from the difference between the total GSH determined and the GSH generated from oxidized glutathione in samples treated with 2-vinylpyridine. The GSH concentration can be determined from an endpoint reading of the color developed at 405 nm or by measuring the rate of color development at 405 nm.

*Erythrocyte antioxidant enzyme activities*

The hemoglobin (Hg) concentration in blood samples was determined with a colorimetric cyanmethemoglobin method using Sigma Diagnostic Drabkin reagents. The activities of the antioxidant enzymes CAT, GR, GPx, and SOD were assayed in lysed erythrocytes by spectrophotometric-specific methods using a BIO-TEK Microplate Reader Synergy HT R (BioTek Instruments, Inc., Winooski, Vermont, U.S.A.). CAT activity was measured by assessing the decomposition of hydrogen peroxide in water at 240 nm as previously reported (*Aebi, 1984*) and expressed as kat/g Hb. GR activity is measured by the reduction in oxidized glutathione to reduced glutathione, and the results are expressed as μmol/min∗gHb [8]. GPx activity was assessed by the oxidized glutathione produced in the reaction at 340 nm and expressed as μmol/min∗gHb [9]. SOD activity was determined by dichromatic analysis (415/450 nm) using xanthine and xanthine oxidase to generate superoxide radicals and expressed as U/g Hb [4].

*Determination of plasma antioxidants (retinol, β-carotene, and α-tocopherol)*

Plasma concentrations of retinol, β-carotene, and α-tocopherol were determined by solvent extraction and ultrahigh-pressure liquid chromatography coupled to mass spectrometry (UHPLC–MS) as reported elsewhere [10,11]. Briefly, 100 μL of plasma was extracted with 300 μl of isopropanol and centrifuged at 11,200 × g for 10 min; 5 μL of the supernatant was injected into an ACQUITY UPLCr BEH C18 50 mm x 2.1 mm internal diameter column with a 1.7 μm particle size at 50 °C (Waters Corporation, Milford, MA, USA). Retinol, β-carotene, and α-tocopherol were eluted using 0.1% formic acid in

methanol as the solvent at a flow rate of 0.600 mL/min. Concentrations of plasma antioxidants are expressed in mg/L.

3.5.2.6 Plasma cytokines

Based on previous reports [12–15], relevant molecules previously described as associated with inflammatory processes and cancer cachexia will be selected for their analysis in plasma. Namely, TNF$\alpha$, IL-6, IL-1β, IFN-$\gamma$, IL-4, IL-10, IL-15, IL-1RA, IL-15, IL-1RA, sIL-6R, sTNFRI, sIL-6R, sTNFRI, sTNFRII, human leukemia inhibitory factor (LIF), human ciliary neurotrophic factor (CNTF) and human proteolysis-inducing factor/dermicidin (PIF/DCD) will be analyzed. Plasma cytokine concentrations will be determined using the Luminex®200™ multianalyte profile analyzer on a Luminex R X MAPTM Technology, LabscanTM 100, (Luminex Corporation, Austin, TX, USA) and the MILLIPLEX® MAP Human High Sensitivity T-Cell Magnetic Bead Panel, Kit Cat. # HSTCMAG-28SK, Kit HCYTA-60K, Kit HSCRMAG-32K-03, Kit HCYP2MAG-62K-01 and Kit HPTP1MAG-66K-01 (MILLIPLEX MAP Multiplex Assay, EMD Millipore Corp, Billerica, MA, USA). Data will be analyzed using the 3.1 version of the xPONENT software (Millipore). The technique has an overall intra- and intraassay coefficient of variation of 10 and 15%, respectively.

3.5.2.7 Plasma metabolomics analysis.

For metabolomic analysis, three plasma aliquots of 0.5 mL each will be used. A multiplex strategy (GlobalMet from EureCat) will be used for the separation and evaluation of both water-soluble and fat-soluble plasma metabolites (lipidomics) based on semitargeted analysis by GLC–MS (plasma metabolome analysis, nontargeted analysis by UPLC–MS and targeted analysis by UPLC–MS) [16–18].

*3.2 Saliva and stool microbiota*

3.6.1 Saliva sampling

The saliva samples obtained during the tests for taste and odor determination were poured into OMNIgene Oral OM-501 (DNA Genotek Inc. Ottawa, Ontario, Canada) tubes containing a bacterial DNA stabilizer and kept at 15-30 °C until they were frozen, in a period not exceeding 24 h, at -80 °C for subsequent transport with dry ice to the analysis site.

3.6.2 Stool sampling

Stool samples were coded and obtained according to the International Human Microbiome Standards (IHMS) guidelines (SOP 01 V2 and SOP-04 V2). Samples are

collected directly by study participants at home using the OMNIgene Gut OM-200 (DNA Genotek Inc., Ottawa, Ontario, Canada) and stored immediately at -20 °C until transported on dry ice and immediately frozen at -80 °C in plastic tubes.

#### 3.6.3 Flow cytometry

Microbial loads from saliva and fecal samples will be processed and analyzed by flow cytometry following a standardized procedure [19]. Briefly, 0.2 g aliquots will be suspended in physiological solution to a total volume of 100 mL (8.5 g l-1 NaCl, VWR); the suspension is diluted 1,000-fold; and samples are filtered using a sterile syringe filter (pore size 5 µm, Sartorius). Next, 1 ml of the microbial cell suspension was stained with 1 µl of SYBR Green I (1:100 dilution in DMSO, 15 min incubation at 37 °C, 10,000 concentrates, Thermo Fisher Scientific). Flow cytometry analysis was performed using a Symphony A5 flow cytometer (Becton Dickison Biosciences). Events are monitored using FL1 533/30-nm and FL3 >670-nm detectors. Instrument and gate settings are kept identical for all samples (fixed staining/gate strategy), and cell counts are converted to microbial loads per ml of saliva or per gram of fecal matter (microbial load index) [20].

#### 3.6.4 Fecal DNA amplification and sequencing

Samples will be processed according to International Human Microbiome Standards (IHMS) guidelines (SOP-07 V2) using a QIAGEN QIAmp PowerFecal ProDNA Kit. DNA amplification will be performed by PCR of the 16S rRNA gene using bacterial-specific primers and adapter-binding barcoding (library preparation). Bacterial DNA sequencing will be analyzed following sequencing standards (IHMS SOP 08, 09 and 10 V1), and sequencing data will be further analyzed for data analysis standards (IHMS SOPs 11).

Sequencing of bacterial DNA from both saliva and feces will be performed with a nanopore procedure (MinION™) that enables direct reading of long DNA sequences [21,22]. Complete amplicons corresponding to the amplification of the bacterial 16S rRNAr gene will be analyzed. Subsequently, during bioinformatics analysis, only amplicons of a desired range can be selected (most reads are approximately 1.5 Kb), which avoids the bias that would be produced by analyzing amplicons of a few hundred bases. By sequencing the complete 16S rRNA gene, both the hypervariable regions (V1 to V9) and constants of the gene are analyzed, which allows greater reliability in the taxonomic characterization of the microbial community under study. In short, the reading of long sequences using nanopores provides a more precise analysis of the microbiological profile of interest compared to other classical technologies that analyze shorter reads, including the techniques known as NGS [22,23].

Briefly, PCR amplification of 16S rRNA genes was performed using the KAPA2G™ Robust HotStart ReadyMix PCR Kit (Kapa Biosystems, Wilmington, MA, USA) in a total volume of 25 µl containing internal primer pairs (50 nM each) and the barcoded external primer mix (3%) from the PCR Barcoding Kit (SQK-PBK004; Oxford Nanopore Technologies, Oxford, UK). Amplification was performed with the following PCR conditions: initial denaturation at 95 °C for 3 min, 5 cycles of 95 °C for 15 s, 55 °C for 15 s and 72 °C for 30 s, 30 cycles of 95 °C for 15 s, 62 °C for 15 s and 72 °C for 30 s, followed by a final extension at 72 °C for 1 min. The amplified DNA was purified with AMPure® XP (Beckman Coulter) and quantified with a NanoDrop® 1000 (Thermo Fischer Scientific, Waltham, MA, USA). A total of 100 ng of DNA was incubated with 1 µl of rapid adapter at room temperature for 5 min. The prepared DNA library (11 µl) was mixed with 34 µl of sequencing buffer, 25.5 µl of loading beads and 4.5 µl of water, loaded onto the R9.4 flow cell (FLO-MIN106; Oxford Nanopore Technologies) and sequenced on MinION™ Mk1B (Matsuo et al., 2021).

MINKNOW 1.11.5 software (Oxford Nanopore Technologies) was used for sequencing data acquisition and chimera removal, as well as for merging direct and reverse reads. The taxonomy will be assigned to ASVs using the classify sklearn naïve Bayes taxonomy classifier (via q2-feature-classifier) [24] against SILVA 16S V3-V4 v132_99 [25] with a similarity threshold of 99%.

#### 3.6.5 Saliva and stool metagenomics

Potential functional profiles (metagenomic analysis) for the sequenced samples will be predicted using PICRUSt2 [26]. Briefly, phylotypes are placed in a reference tree containing 20,000 complete 16S rRNA genes from prokaryotic genomes in the Integrated Microbial Genomes (IMG) database. Functional annotation of these genomes is based on the Clusters of Orthologous Groups of proteins (COG) and Enzyme Commission numbers (EC) databases. To infer Metacyc metabolic pathways, EC numbers are first regrouped into MetaCyc reactions. Pathway abundances will be calculated as the harmonic mean of the abundances of the key reactions in each sample. To infer the abundance of each gene family per sample, phylotype abundances will be corrected for their 16S rRNA gene copy number and then multiplied by their functional predictions [27].

## References

1. Lepage, G.; Roy, C.C. Specific Methylation of Plasma Nonesterified Fatty Acids in a One-Step Reaction. *J Lipid Res* **1988**, *29*, 227–235.
2. de la Torre-Aguilar, M.J.; Gomez-Fernandez, A.; Flores-Rojas, K.; Martin-Borreguero, P.; Mesa, M.D.; Perez-Navero, J.L.;

Olivares, M.; Gil, A.; Gil-Campos, M. Docosahexaenoic and Eicosapentaenoic Intervention Modifies Plasma and Erythrocyte Omega-3 Fatty Acid Profiles But Not the Clinical Course of Children With Autism Spectrum Disorder: A Randomized Control Trial. *Front. Nutr.* **2022**, *9*, doi:10.3389/FNUT.2022.790250.

3. Miles, E.A.; Noakes, P.S.; Kremmyda, L.S.; Vlachava, M.; Diaper, N.D.; Rosenlund, G.; Urwin, H.; Yaqoob, P.; Rossary, A.; Farges, M.C.; et al. The Salmon in Pregnancy Study: Study Design, Subject Characteristics, Maternal Fish and Marine n-3 Fatty Acid Intake, and Marine n-3 Fatty Acid Status in Maternal and Umbilical Cord Blood. *Am. J. Clin. Nutr.* **2011**, *94*, doi:10.3945/AJCN.110.001636.
4. García-Rodríguez, C.E.; Mesa, M.D.; Olza, J.; Vlachava, M.; Kremmyda, L.S.; Diaper, N.D.; Noakes, P.S.; Miles, E.A.; Ramírez-Tortosa, M.C.; Liaset, B.; et al. Does Consumption of Two Portions of Salmon per Week Enhance the Antioxidant Defense System in Pregnant Women? *Antioxid. Redox Signal.* **2012**, *16*, 1401–1406, doi:10.1089/ARS.2012.4508.
5. García-Rodríguez, C.E.; Helmersson-Karlqvist, J.; Dolores Mesa, M.; Miles, E.A.; Noakes, P.S.; Vlachava, M.; Kremmyda, L.S.; Diaper, N.D.; Godfrey, K.M.; Calder, P.C.; et al. Does Increased Intake of Salmon Increase Markers of Oxidative Stress in Pregnant Women? The Salmon in Pregnancy Study. *Antioxid. Redox Signal.* **2011**, *15*, 2819–2823, doi:10.1089/ARS.2011.4108.
6. Garcia-Rodriguez, C.E.; Olza, J.; Mesa, M.D.; Aguilera, C.M.; Miles, E.A.; Noakes, P.S.; Vlachava, M.; Kremmyda, L.S.; Diaper, N.D.; Godfrey, K.M.; et al. Fatty Acid Status and Antioxidant Defense System in Mothers and Their Newborns after Salmon Intake during Late Pregnancy. *Nutrition* **2017**, *33*, 157–162, doi:10.1016/J.NUT.2016.05.015.
7. Re, R.; Pellegrini, N.; Proteggente, A.; Pannala, A.; Yang, M.; Rice-Evans, C. Antioxidant Activity Applying an Improved ABTS Radical Cation Decolorization Assay. *Free Radic. Biol. Med.* **1999**, *26*, 1231–1237, doi:10.1016/S0891-5849(98)00315-3.
8. Carlberg, I.; Mannervik, B. Glutathione Reductase. *Methods Enzymol.* **1985**, *113*, 484–490, doi:10.1016/S0076-6879(85)13062-4.
9. Flohé, L.; Günzler, W.A. Assays of Glutathione Peroxidase. *Methods Enzymol.* **1984**, *105*, 114–120, doi:10.1016/S0076-6879(84)05015-1.
10. Battino, M.; Leone, L.; Bompadre, S. High-Performance Liquid Chromatography-EC Assay of Mitochondrial Coenzyme Q9, Coenzyme Q9H2, Coenzyme Q10, Coenzyme Q10H2, and Vitamin E with a Simplified On-Line Solid-Phase Extraction. *Methods Enzymol.* **2004**, *378*, 156–162, doi:10.1016/S0076-6879(04)78012-X.
11. Ordóñez-Díaz, M.D.; Gil-Campos, M.; Flores-Rojas, K.; Muñoz-Villanueva, M.C.; Mesa, M.D.; de la Torre-Aguilar, M.J.; Gil, Á.; Pérez-Navero, J.L. Impaired Antioxidant Defence Status Is Associated With Metabolic-Inflammatory Risk Factors in Preterm Children With Extrauterine Growth Restriction: The BIORICA Cohort Study. *Front. Nutr.* **2021**, *8*, doi:10.3389/FNUT.2021.793862.
12. Argilés, J.M.; Busquets, S.; Stemmler, B.; López-Soriano, F.J. Cancer Cachexia: Understanding the Molecular Basis. *Nat. Rev. Cancer* **2014**, *14*, 754–762, doi:10.1038/NRC3829.
13. Argilés, J.M.; López-Soriano, F.J.; Stemmler, B.; Busquets, S. Cancer-Associated Cachexia - Understanding the Tumour Macroenvironment and Microenvironment to Improve Management. *Nat. Rev. Clin. Oncol.* **2023**, *20*, doi:10.1038/S41571-023-00734-5.
14. Mendes, M.C.S.; Pimentel, G.D.; Costa, F.O.; Carvalheira, J.B.C. Molecular and Neuroendocrine Mechanisms of Cancer Cachexia. *J. Endocrinol.* **2015**, *226*, R29–R43, doi:10.1530/JOE-15-0170.
15. Tisdale, M.J. Are Tumoral Factors Responsible for Host Tissue Wasting in Cancer Cachexia? *Future Oncol.* **2010**, *6*, 503–513, doi:10.2217/FON.10.20.

16. Rangel-Huerta, O.D.; Pastor-Villaescusa, B.; Gil, A. Are We Close to Defining a Metabolomic Signature of Human Obesity? A Systematic Review of Metabolomics Studies. *Metabolomics* **2019**, *15*, doi:10.1007/S11306-019-1553-Y.
17. Rangel-Huerta, O.D.; Gil, A. Nutrimetabolomics: An Update on Analytical Approaches to Investigate the Role of Plant-Based Foods and Their Bioactive Compounds in Non-Communicable Chronic Diseases. *Int. J. Mol. Sci.* **2016**, *17*, doi:10.3390/IJMS17122072.
18. Rangel-Huerta, O.D.; Gomez-Fernández, A.; de la Torre-Aguilar, M.J.; Gil, A.; Perez-Navero, J.L.; Flores-Rojas, K.; Martín-Borreguero, P.; Gil-Campos, M. Metabolic Profiling in Children with Autism Spectrum Disorder with and without Mental Regression: Preliminary Results from a Cross-Sectional Case-Control Study. *Metabolomics* **2019**, *15*, doi:10.1007/S11306-019-1562-X.
19. Vandeputte, D.; Kathagen, G.; D'Hoe, K.; Vieira-Silva, S.; Valles-Colomer, M.; Sabino, J.; Wang, J.; Tito, R.Y.; De Commer, L.; Darzi, Y.; et al. Quantitative Microbiome Profiling Links Gut Community Variation to Microbial Load. *Nature* **2017**, *551*, 507–511, doi:10.1038/NATURE24460.
20. Prest, E.I.; Hammes, F.; Kötzsch, S.; van Loosdrecht, M.C.M.; Vrouwenvelder, J.S. Monitoring Microbiological Changes in Drinking Water Systems Using a Fast and Reproducible Flow Cytometric Method. *Water Res.* **2013**, *47*, 7131–7142, doi:10.1016/J.WATRES.2013.07.051.
21. Nygaard, A.B.; Tunsjø, H.S.; Meisal, R.; Charnock, C. A Preliminary Study on the Potential of Nanopore MinION and Illumina MiSeq 16S RRNA Gene Sequencing to Characterize Building-Dust Microbiomes. *Sci. Rep.* **2020**, *10*, 3209, doi:10.1038/S41598-020-59771-0.
22. Matsuo, Y.; Komiya, S.; Yasumizu, Y.; Yasuoka, Y.; Mizushima, K.; Takagi, T.; Kryukov, K.; Fukuda, A.; Morimoto, Y.; Naito, Y.; et al. Full-Length 16S RRNA Gene Amplicon Analysis of Human Gut Microbiota Using MinION™ Nanopore Sequencing Confers Species-Level Resolution. *BMC Microbiol.* **2021**, *21*, doi:10.1186/S12866-021-02094-5.
23. de Siqueira, G.M.V.; Pereira-dos-Santos, F.M.; Silva-Rocha, R.; Guazzaroni, M.E. Nanopore Sequencing Provides Rapid and Reliable Insight Into Microbial Profiles of Intensive Care Units. *Front. public Heal.* **2021**, *9*, doi:10.3389/FPUBH.2021.710985.
24. Bokulich, N.A.; Kaehler, B.D.; Rideout, J.R.; Dillon, M.; Bolyen, E.; Knight, R.; Huttley, G.A.; Gregory Caporaso, J. Optimizing Taxonomic Classification of Marker-Gene Amplicon Sequences with QIIME 2's Q2-Feature-Classifier Plugin. *Microbiome* **2018**, *6*, doi:10.1186/S40168-018-0470-Z.
25. Quast, C.; Pruesse, E.; Yilmaz, P.; Gerken, J.; Schweer, T.; Yarza, P.; Peplies, J.; Glöckner, F.O. The SILVA Ribosomal RNA Gene Database Project: Improved Data Processing and Web-Based Tools. *Nucleic Acids Res.* **2013**, *41*, doi:10.1093/NAR/GKS1219.
26. Douglas, G.M.; Maffei, V.J.; Zaneveld, J.R.; Yurgel, S.N.; Brown, J.R.; Taylor, C.M.; Huttenhower, C.; Langille, M.G.I. PICRUSt2 for Prediction of Metagenome Functions. *Nat. Biotechnol.* **2020**, *38*, 685–688, doi:10.1038/S41587-020-0548-6.
27. Plaza-Díaz, J.; Manzano, M.; Ruiz-Ojeda, F.J.; Giron, M.D.; Salto, R.; López-Pedrosa, J.M.; Santos-Fandila, A.; Garcia-Corcoles, M.T.; Rueda, R.; Gil, Á. Intake of Slow-Digesting Carbohydrates Is Related to Changes in the Microbiome and Its Functional Pathways in Growing Rats with Obesity Induced by Diet. *Front. Nutr.* **2022**, *9*, doi:10.3389/FNUT.2022.992682.
