# Supplementary material for: Effect of Regular Consumption of a Miraculin-Based Food Supplement on Taste Perception and Nutritional Status in Malnourished Cancer Patients: A Triple-Blind, Randomized, Placebo-Controlled Clinical Trial-CLINMIR Pilot Protocol
Source: Nutrients. 2023 Nov 1;15(21):4639. doi: 10.3390/nu15214639 (PMC10648678; doi:10.3390/nu15214639)
Supplement: Supplementary file 1 [file nutrients-15-04639-s001.zip › File S4. Detailed Procedures.pdf]

nutrients

MDPI

File S4. Detailed Procedure

Effect of Regular Consumption of a Miraculin-Based Food Supplement on Taste Perception and Nutritional Status in Malnourished Cancer Patients: A Triple-Blind, Randomized, Placebo-Controlled Clinical Trial–CLINMIR Pilot Protocol

4. Detailed Procedure

The clinical trial is divided into two phases (Selection and Intervention Phase), which include a selection visit (v0) and five more visits through the study (Figure 1).

4.1 Selection Phase

4.2.1 Visit 0 (v0, selection visit).

After clinical trial approval by the Ethics Committee, patients undergoing neoadjuvant treatment with chemotherapy or chemoradiotherapy for at least three months and malnutrition who were referred to the Clinical Nutrition and Dietetics Unit from the Oncology Service were invited to participate in the study. If the patient is interested in participating, compliance with the study selection criteria is verified. When all requirements are met, an information-to-read document is provided. Two researchers are in charge of recruiting patients, explaining the protocol and providing the informed consent form. Once the patient's doubts were clarified and if the patient was interested, informed consent was signed.

During this visit, the following activities are carried out:

- Nutritional status assessment (GLIM Criteria)
- Electrogustometry
- Taste strips test
- Taste and Smell Survey Modified

The following documentation is given to the patient to complete visit 1 (v1):

- Food Daily Record (3 days per holiday)
- Food Frequency Questionnaire
- International Physical Activity Questionnaire
- Quality of Life Questionnaire

During this visit, the patient is informed about the next visit date and provided with:

- Blood sample extraction appointment
- Stool container for microbiota and metagenome analysis
- Urine container for 8-iso-PGF2α determination

4.2 Experimental Phase

This phase includes 5 face-to-face visits. Except for visit 1, these visits are conditioned by the patients' chemotherapy treatment sessions and are carried out in the following 4-7 days after their infusion, a period during which there is greater toxicity and taste affectation.

4.2.1 Visit 1 (v1)

Once it was verified that patients met the inclusion and exclusion criteria, the experimental phase's first visit was made. In this visit, the patient is randomized, and the following initial measures are taken:

- Health study (blood pressure and heart rate)
- Morphofunctional assessment:
    - Anthropometric measurements
    - Electrical bioimpedance
    - Dynamometry
    - Nutritional ultrasound
    - Up and Go Test
- Sniffin' Stick Smell Test
- Collection and measurement of saliva volume
- Blood sample extraction coinciding with the prechemotherapy analysis:
    - Biochemical parameters
    - Plasma metabolomic analysis
    - Plasma cytokine profile
    - Fatty acids from the erythrocyte membrane
    - Enzymatic antioxidant defense system in erythrocytes
    - Body oxidative stress in urine
    - Fecal microbiota and metagenome
    - Saliva microbiota and metagenome

During this visit, the following documentation is collected:

- Food Daily Record (3 days per holiday)
- Food consumption frequency
- International Physical Activity Questionnaire

- Quality of life questionnaire
- Feces sample
- Urine sample

Once the tests have been carried out, the patient is instructed on:

- Nutritional treatment. If an oral nutritional supplement is needed, a polymeric, hypercaloric, and hyperproteic formula enriched in omega-3 fatty acids is prescribed depending on their energy requirements.
- Healthy eating guidelines for cancer patients using the book "Cooking with Science Against Cancer" as a support are given to the patient.
- Physical exercise guidelines

During this visit, the study treatment (Miraculin-based food supplement or placebo) is delivered to the patient based on randomization, and the following documentation is delivered to bring the next visit (v2):

- Product efficacy satisfaction questionnaire (prechemotherapy)
- Next face-to-face visit date.

The following documentation is also delivered to bring on the third visit (v3):

- Product consumption control daily sheet
- Product consumption tolerance record sheet
- Record sheet of adverse effects

4.2.2 Visit 2 (v2)

Carried out 4–7 days after the chemotherapy session, during this visit, the following measurements are made:

- Anthropometric measurements
- Electrogustometry
- Smell and taste tests:
  - Taste Strips Test
  - Sniffin 'Sticks Smell Test
- Collection and measurement of saliva volume
- Taste and Smell Modified Survey
- Product Efficacy Satisfaction Questionnaire (Post Chemotherapy)

The following behavioral reinforcements are also carried out on this visit:

- Nutritional treatment and physical activity
- Consumption and registration of the assigned treatment.
- Tolerance and adverse effects registry

At the end of this visit, the following documentation and material are given to the patient. These have to be delivered on the next visit (v3):

- Food Daily Record (3 days per holiday)
- International Physical Activity Questionnaire
- Quality of Life Questionnaire
- Blood sample extraction appointment
- Feces container
- Urine container

4.2.3 Visit 3 (v3)

This visit took place ±1 month after visit 1 (v1) and 3-4 days after the patient's chemotherapy treatment session. During this visit, the following actions are carried out:

- Nutritional status assessment
- Health study (blood pressure and heart rate)
- Morphofunctional assessment:
    - Anthropometric measurements
    - Electrical bioimpedance
    - Dynamometry
    - Up and Go Test
- Electrogustometry
- Smell and taste tests:
    - Taste Strips Test
    - Sniffin 'Sticks Smell Test
- Collection and measurement of saliva volume
- Blood sample extraction coinciding with the prechemotherapy analysis:
    - Biochemical parameters
    - Plasma metabolomic analysis
    - Plasma cytokine profile
    - Fatty acids from the erythrocyte membrane
    - Enzymatic antioxidant defense system in erythrocytes
    - Body oxidative stress in urine
    - Fecal microbiota and metagenome
    - Saliva microbiota and metagenome

- Product efficacy satisfaction questionnaire

During this visit, the following documentation is collected:

- Food Daily Record (3 days per holiday)
- International Physical Activity Questionnaire
- Quality of Life Questionnaire
- Product consumption control daily sheet
- Product consumption tolerance record sheet
- Record sheet of adverse effects
- Feces sample
- Urine sample

The following behavioral reinforcements are also carried out on this visit:

- Nutritional treatment and physical activity
- Consumption and registration of the assigned treatment.
- Tolerance and adverse effects registry

At the end of this visit, the following documentation and material are given to the patient. These had to be delivered on the next visit (v4):

- Next face-to-face visit date
- Treatment product based on randomization
- Blood sample extraction appointment
- Food Daily Record (3 days per holiday)
- International Physical Activity Questionnaire
- Quality of Life Questionnaire
- Product consumption control daily sheet
- Product consumption tolerance record sheet
- Record sheet of adverse effects

4.2.4 Visit 4 (v4)

This visit took place ±2 months after visit 1 (v1) and 3-4 days after the patient's chemotherapy treatment session. During this visit, the following actions are carried out:

- Nutritional status assessment
- Health study (blood pressure and heart rate)
- Morphofunctional assessment:
    - Anthropometric measurements
    - Electrical bioimpedance

    - Dynamometry
    - Up and Go Test
- Electrogustometry
- Smell and taste tests:
    - Taste Strips Test
    - Sniffin' Sticks Smell Test
- Collection and measurement of saliva volume
- Blood sample extraction coinciding with the prechemotherapy analysis:
    - Biochemical parameters
    - Plasma metabolomic analysis
    - Plasma cytokine profile
    - Fatty acids from the erythrocyte membrane
    - Enzymatic antioxidant defense system in erythrocytes
    - Body oxidative stress in urine
    - Fecal microbiota and metagenome
    - Saliva microbiota and metagenome

During this visit, the following documentation is collected:

- Food Daily Record (3 days, one holiday)
- International Physical Activity Questionnaire
- Quality of Life Questionnaire
- Product consumption control daily sheet
- Product consumption tolerance record sheet
- Record sheet of adverse effects

The following behavioral reinforcements are also carried out on this visit:

- Nutritional treatment and physical activity
- Consumption and registration of the assigned treatment.
- Tolerance and adverse effects registry

At the end of this visit, the following documentation and material are given to the patient. These have to be delivered on the next visit (v5):

- Next face-to-face visit date
- Treatment product based on randomization
- Food Daily Record (3 days per holiday)
- Food consumption frequency
- International Physical Activity Questionnaire

- Quality of Life Questionnaire
- Product consumption control daily sheet
- Product consumption tolerance record sheet
- Record sheet of adverse effects
- Blood sample extraction appointment
- Feces sample
- Urine sample

4.2.5 Visit 5 (v5)

This visit took place ±3 months after visit 1 (v1) and 3-4 days after the patient's chemotherapy treatment session. During this visit, the following actions are carried out:

- Nutritional status assessment
- Health study (blood pressure and heart rate)
- Morphofunctional assessment:
  - Anthropometric measurements
  - Electrical bioimpedance
  - Dynamometry
  - Nutritional ultrasound
  - Up and Go Test
- Electrogustometry
- Taste and Smell Survey Modified
- Smell and taste tests:
  - Taste Strips Test
  - Sniffin' Sticks Smell Test
- Collection and measurement of saliva volume
- Blood sample extraction coinciding with the prechemotherapy analysis:
  - Biochemical parameters
  - Plasma metabolomic analysis
  - Plasma cytokine profile
  - Fatty acids from the erythrocyte membrane
  - Enzymatic antioxidant defense system in erythrocytes
  - Body oxidative stress in urine
  - Fecal microbiota and metagenome
  - Saliva microbiota and metagenome
- Product efficacy satisfaction questionnaire

During this visit, the following documentation is collected:

- Food Daily Record (3 days, one holiday)
- Food consumption frequency
- International Physical Activity Questionnaire
- Quality of Life Questionnaire
- Product consumption control daily sheet
- Product consumption tolerance record sheet
- Record sheet of adverse effects
- Feces sample
- Urine sample

The following behavioral reinforcements are also carried out on this visit:

- Nutritional treatment and physical activity
